# Twenty-five years of experience with patient-reported outcome measures in soft-tissue sarcoma patients: a systematic review

**DOI:** 10.1007/s11136-024-03755-4

**Published:** 2024-09-11

**Authors:** Jasmijn D. Generaal, Marnix R. Jansen, Goudje L. van Leeuwen, Robert J. van Ginkel, Lukas B. Been, Barbara L. van Leeuwen

**Affiliations:** 1 Division of Surgical Oncology, Department of Surgery, University Medical Center Groningen, University of Groningen, Hanzeplein 1, 9713 GZ Groningen, The Netherlands; 2 Division of Vascular Surgery, Department of Surgery, University Medical Center Groningen, University of Groningen, Hanzeplein 1, 9713 GZ Groningen, The Netherlands

**Keywords:** Soft-tissue sarcoma, Patient-reported outcome measure, Patient-reported outcome, Consensus-based Standards for the Selection of Health Measurement Instruments, COSMIN, Review

## Abstract

**Purpose:**

As the importance of the patient’s perspective on treatment outcome is becoming increasingly clear, the availability of patient-reported outcome measures (PROMs) has grown accordingly. There remains insufficient information regarding the quality of PROMs in patients with soft-tissue sarcomas (STSs). The objectives of this systematic review were (1) to identify all PROMs used in STS patients and (2) to critically appraise the methodological quality of these PROMs.

**Methods:**

Literature searches were performed in MEDLINE and Embase on April 22, 2024. PROMs were identified by including all studies that evaluate (an aspect of) health-related quality of life in STS patients by using a PROM. Second, studies that assessed measurement properties of the PROMs utilized in STS patients were included. Quality of PROMs was evaluated by performing a COSMIN analysis.

**Results:**

In 59 studies, 39 PROMs were identified, with the Toronto Extremity Salvage Score (TESS) being the most frequently utilized. Three studies evaluated methodological quality of PROMs in the STS population. Measurement properties of the TESS, Quick Disability of the Arm, Shoulder and Hand (QuickDASH) and European Organization for Research and Treatment for Cancer Quality of Life Questionnaire (EORTC-QLQ-C30) were reported. None of the PROMs utilized in the STS population can be recommended for use based on the current evidence and COSMIN analysis.

**Conclusion:**

To ensure collection of reliable outcomes, PROMs require methodological evaluation prior to utilization in the STS population. Research should prioritize on determining relevant content and subsequently selecting the most suitable PROM for assessment.

**Supplementary Information:**

The online version contains supplementary material available at 10.1007/s11136-024-03755-4.

## Introduction

Sarcomas are rare malignancies originating from mesenchymal tissues and can be divided in bone and soft-tissue sarcomas (STSs). The incidence of STSs is 4–5/100,000/year in Europe [1] and increasing in the ageing population [2]. Median age at presentation is 65 years [1, 3]. There is a range of clinical presentations for STSs, which challenges specialists to provide optimal patient care. Treatment therefore takes place in medical centers with a specialized multidisciplinary tumor board [4] and consists of a patient-tailored combination of surgical resection, radiotherapy (RT) and/or chemotherapy [1]. All of these treatments potentially cause treatment-related morbidity, for instance impaired wound healing, stiffness, pain and reduced mobility [5–8].

Research in the STS population has focused on multimodality treatments to achieve optimal oncological outcomes while striving to reduce treatment-related morbidity. Among other achievements, this has led to a shift in the timing of RT from postoperative to preferably preoperative, employing smaller radiation fields and lower total RT dosages [9]. In most studies, treatment-related morbidity is defined as functional outcome following surgery. Functional outcome can be determined from the doctors’ perspective, but increased awareness of the value of the patient’s perspective has resulted in the utilization of patient-reported outcome measures (PROMs) in STS patients [8]. PROMs are questionnaires reported directly by the patient, without interpretation of the patient’s response by a clinician or anyone else [10]. PROMs serve as tools for assessing patient-reported outcomes (PROs), which may pertain to different aspects of health-related quality of life, such as functional status or mental wellbeing, or offer a comprehensive evaluation of health-related quality of life.

One of the most widely used PROMs to measure outcome in patients with musculoskeletal tumors is the Toronto Extremity Salvage Score (TESS) [11], which was developed in 1996. In addition to functional status, STS diagnosis and subsequent interventions impact other aspects of the multidimensional concept health-related quality of life, as elucidated by Wilson and Cleary [12]. Various generic [13–15] and disease-specific [16–18] PROMs have been employed to evaluate (aspects of) health-related quality of life of STS patients. While selecting a reliable and valid PROM can be challenging due to the available options, it remains indispensable since understanding the diverse dimensions of health-related quality of life serves as the cornerstone of value-based healthcare [19].

The primary objective of this systematic review was to identify all PROMs utilized in the STS population. Secondarily, we aimed to methodologically evaluate the quality of these PROMs. By methodologically assessing PROMs, our goal was to determine their suitability in accurately capturing the experiences and outcomes of STS patients.

## Methods

This systematic review was conducted in accordance with the Preferred Reporting Items for Systematic Reviews and Meta-Analyses (PRISMA) statement [20] and the Consensus-based Standards for the Selection of Health Measurement Instruments (COSMIN) guidelines [21, 22] The PRISMA checklist can be found in Supplementary Information 1.

### Literature search

Two searches were conducted: one to identify all PROMs utilized in the STS population and another to find all studies reporting on measurement properties of these PROMs.

#### Eligibility criteria

For the identification of PROMs, all studies that involved (a) STS patients and evaluated (b) an aspect of health-related quality of life or health-related quality of life comprehensively by using a PROM were included for analysis. Studies were excluded when using a PROM as outcome measurement in a trial randomizing treatment and including patients younger than 18 years old. Studies utilizing PROMs in both STS and bone sarcoma patients were excluded if the results were not analyzed separately for each entity, along with studies not published in English.

According to the COSMIN guidelines, eligibility criteria for studies on the quality of PROMs include: (a) the target population, (b) the name(s) of the PROMs and (c) the evaluation of measurement properties. For finding studies that report on measurement properties, studies were considered eligible if they involved (a) STS patients, included (b) at least one of the PROMs used in the STS population and evaluated (c) at least one measurement property. Studies were excluded if they included both bone and STS patients for analysis due to significant clinical differences between these patient populations [1, 23], which would compromise the validity and reliability of measurement property assessments. Additionally, studies were excluded if full-text English was unavailable.

#### Search strategies

The search strategies were composed as follows:

- The target population was defined as STS patients.
- To identify all (names of the) PROMs used in the STS population, a separate search was conducted that consisted of (a) the target population and (b) the constructs of interest. This was defined as (a) STS patients and (b) (aspects of) health-related quality of life, with the same (c) exclusion filter as specified below (d).
- Terwee et al. [24] developed a search filter capable of identifying all measurement properties.
- The exclusion filter by Terwee et al. [24] was applied to eliminate irrelevant records from the searches, such as animal studies and conference abstracts.

**Fig. 1:**
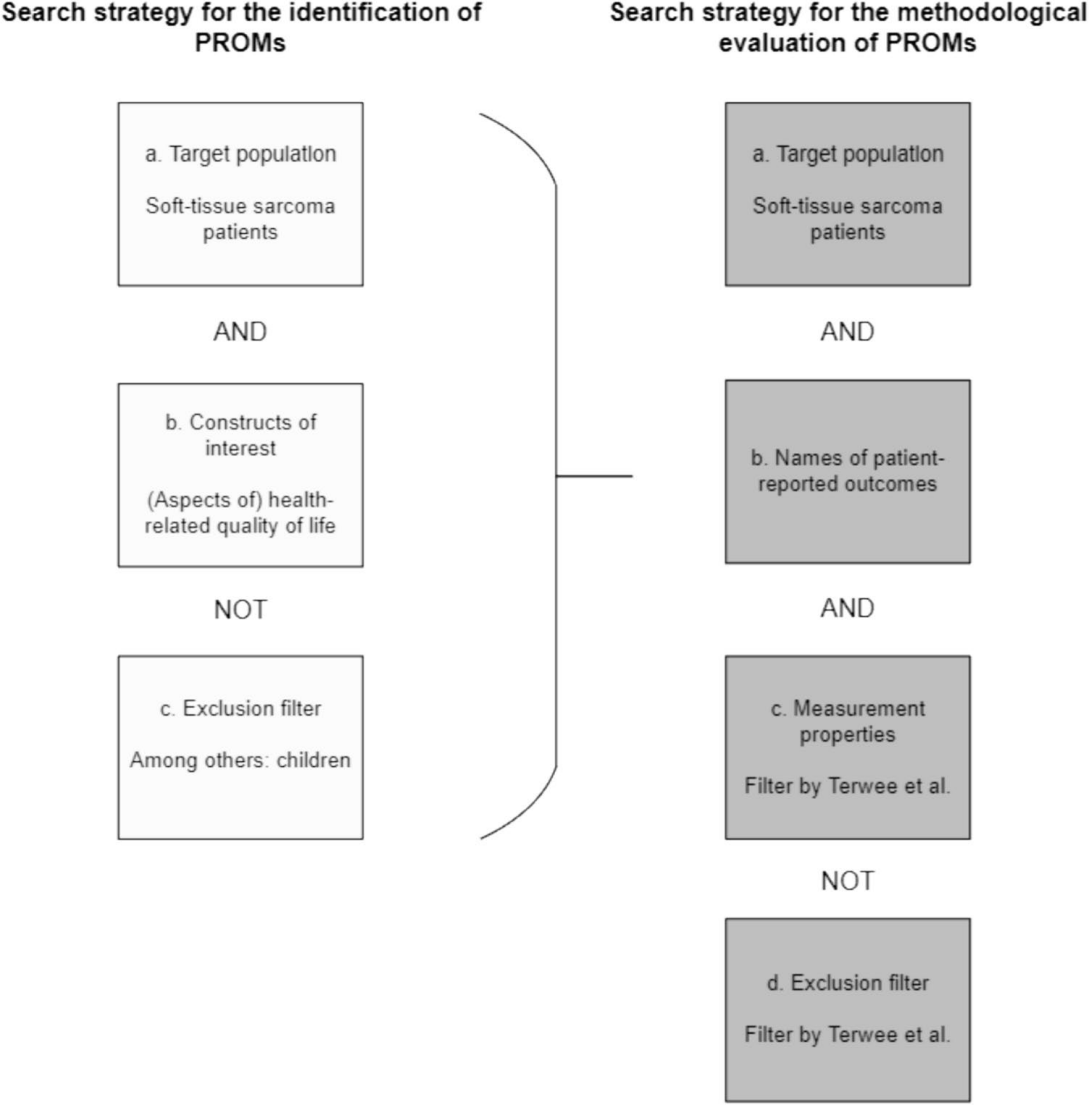
Overview of the composition of the search strategy for the identification of PROMs (search 1) and the search strategy for the methodological evaluation of PROMs (search 2)

Refer to Fig. 1 for a schematic depiction outlining the composition of the searches. Supplementary Information 7 delineates the search strategy for the identification of PROMs (search 1) and the search strategy for the methodological evaluation of PROMs (search 2) employed in MEDLINE. Supplementary Information 2 provides the search strategies in Embase.

#### Information sources

MEDLINE and Embase were searched on February 2nd 2023 and June 28th 2023. Search 1 took place on February 2nd 2023, whereas search 2 took place June 28th 2023. Both searches were updated on April 22nd 2024. Search strategies were composed by a senior librarian. After search 1, citation tracking was performed by reviewing reference lists of all reviews on PROs in the sarcoma population for eligible reports by hand. These reviews were identified using the elements (sarcoma) AND (patient-reported outcome), using the filters systematic review and review. Following search 2, the reference lists of all included studies were reviewed. No time limits or language restrictions were applied to the searches.

### Selection process

For both searches, two reviewers (MRJ and JDG) independently assessed all records for eligibility. In cases of disagreement, consensus on which articles to screen full-text was achieved through discussion. If needed, a third reviewer (BvL) was consulted to make final decisions. The identical process was reiterated during the full-text review for inclusion. The searches were updated subsequent to the submission of this manuscript. A fourth reviewer, GvL, took over the role previously held by MRJ.

### Data collection

All data were collected by one reviewer (JDG), with a second reviewer (MRJ) independently collecting data from 10% of randomly selected studies to check for discrepancies. Inter-rater reliability, assessed using Cohen’s kappa, was 0.88. Following the updated search, GvL assumed MRJ’s role, resulting in a Cohen’s kappa of 0.94. First, the data collection process took place for the studies included after search 1. By conducting data collection for these studies initially, all names of the PROMs utilized in the STS population were identified, facilitating search 2 aimed at finding studies on measurement properties of PROMs in the STS population. Relevant study and PROM characteristics were extracted using a data collection form.

#### Identification of patient-reported outcome measures

The author, title, year, and source of publication of the articles were recorded. Additionally, the study design, inclusion period, and specific characteristics of the patient population (such as stage of disease, age, and gender of patients) were extracted. The primary endpoint of the study, timing of evaluation using PROM(s), name and type of PROM used in the study, and corresponding construct were also reported.

#### Characteristics of patient-reported outcome measures

The first reference (development study) of each PROM used in the STS population was retrieved. The construct evaluated by the PROM was determined and it was reported whether the PROM was generic or disease-specific. Information such as the number of items, rating scale, item score, and total score were extracted from each PROM and recorded in the data collection form.

### Risk of bias

Risk of bias of the studies included after search 1 was evaluated using the National Institutes of Health (NIH) Quality Assessment Tool of Observational Cohort and Cross-sectional Studies [25, 26]. This involved answering 11 questions on methodology and rating the overall quality of each study. Question 10, 11 and 12 were deemed not applicable to the studies. Question 10 assesses repeated exposure measurement, which was not applicable since the exposure was either STS diagnosis or treatment, which would not change over time. Question 11 evaluates bias in outcome measures, which aligns with the objective of the systematic review. Question 12 pertains to blinding of outcome assessors, which was not applicable as outcome measures were patient-reported. Risk of bias assessment was conducted independently by two reviewers (MRJ and JDG). Any discrepancies in assessment were resolved through discussion, with a third reviewer (BvL) making final decisions if necessary. Following the updated search, GvL assumed MRJ’s role.

### Methodological assessment of patient-reported outcome measures

The COSMIN database for systematic reviews on outcome measurement instruments was searched; no previous systematic reviews on the measurement properties of PROMs in the STS population were found.

The COSMIN manual defines multiple measurement properties, divided in the domains reliability, validity and responsiveness [21]. For each included study identified through the second search, the evaluated measurement properties were determined. It is possible for multiple measurement properties of a single PROM to be evaluated within one study. These identified measurement properties were assessed individually.

Content validity, considered the most important measurement property of a PROM, is defined as the degree to which an instrument is an adequate reflection of the construct to be measured [22]. Quality of evaluation of content validity was assessed by using a separate manual; the COSMIN manual for assessing content validity [22]. Content validity was assessed in the review by (a) appraising the development quality of PROMs which were developed involving STS patients and (b) evaluating the quality of content validity studies conducted in STS patients. For all included PROMs, the development study was retrieved. If these studies involved STS patients, the quality was evaluated using the standards for evaluating the quality of the PROM design (item generation) and standards for evaluating the quality of a cognitive interview study or other pilot test. For available content validity studies in the STS population, quality was assessed by rating five parts: asking patients about relevance of the PROM; asking patients about the comprehensiveness of the PROM; asking patients about the comprehensibility of the PROM; asking professionals about the relevance of the PROM and asking professionals about the comprehensiveness of the PROM.

For all measurement properties other than content validity, the methodological quality of each assessment was established using the COSMIN risk of bias checklist [21]. Methodological flaws were evaluated by assigning ratings to standards for each measurement property (very good, adequate, doubtful, or inadequate). The overall quality was determined by the lowest rating among all standards in the checklist. In addition, data concerning the study population, disease characteristics, instrument administration and interpretability were gathered from the included studies. Subsequently, the study results were compared with the criteria for good measurement properties as outlined in the COSMIN manual. These were rated as sufficient (+), insufficient (−) or indeterminate (?). See Table 1 for an overview of the criteria for good measurement properties.

**Table 1:** Criteria for good measurement properties

Measurement property	Rating^a^	Criteria
Structural validity	+	**CTT** CFA: CFI or TLI or comparable measure > 0.95 OR RMSEA < 0.06 OR SRMR < 0.08 **IRT/Rasch** No violation of unidimensionality: CFI or TLI or comparable measure > 0.95 OR RMSEA < 0.06 OR SRMR < 0.08 AND No violation of local independence: residual correlations among the items after controlling for the dominant factor < 0.20 OR Q3’s < 0.37 AND No violation of monotonicity: adequate looking graphs OR item scalability > 0.30 AND Adequate model fit: IRT: χ^2^ > 0.01 Rasch: infit and outfit mean squares ≥ 0.5 and ≤ 1.5 OR Z-standardized values > − 2 and < 2
	?	**CTT**: Not all information for ‘+’ reported **IRT/Rasch**: Model fit not reported
	−	Criteria for ‘+’ not met
Internal consistency	+	At least low evidence for sufficient structural validity AND Cronbach’s alpha(s) ≥ 0.70 for each unidimensional scale or subscale
	?	Criteria for “at least low evidence for sufficient structural validity not met”
	−	At least low evidence for sufficient structural validity AND Cronbach’s alpha(s) < 0.70 for each unidimensional scale or subscale
Reliability	+	ICC or weighted Kappa ≥ 0.70
	?	ICC or weighted Kappa not reported
	−	ICC or weighted Kappa < 0.70
Measurement error	+	SDC or LoA < MIC
	?	MIC not defined
	−	SDC or LoA > MIC
Hypotheses testing for construct validity	+	The result is in accordance with the hypothesis
	?	No hypothesis defined
	−	The result is not in accordance with the hypothesis
Cross-cultural validity/measurement invariance	+	No important differences found between group factors (such as age, gender, language) in multiple group factor analysis OR no important DIF for group factors (McFadden’s R^2^ < 0.02)
	?	No multiple group analysis OR DIF analysis performed
	−	Important differences between group factors OR DIF was found
Criterion validity	+	Correlation with gold standard ≥ 0.70 OR AUC ≥ 0.70
	?	Not all information for ‘+’ reported
	−	Correlation with gold standard < 0.70 OR AUC < 0.70
Responsiveness	+	The result is in accordance with the hypothesis OR AUC ≥ 0.70
	?	No hypothesis defined
	−	The result is not in accordance with the hypothesis OR AUC < 0.70

^a^“+” = sufficient, “?” = indeterminate, “−” = insufficient

*CTT* classical test theory, *CFA* confirmatory factor analysis, *CFI* comparative fit index, *TLI* Trucker-Lewis Index, *RMSEA* root mean square error of approximation, *SRMR* standardized root mean residuals, *IRT* item response theory, *ICC* intraclass correlation coefficient, *SDC* smallest detectable change, *LoA* limits of agreement, *MIC* minimal important change, *DIF* differential item functioning, *AUC* area under the curve

## Results

### Study selection

**Fig. 2:**
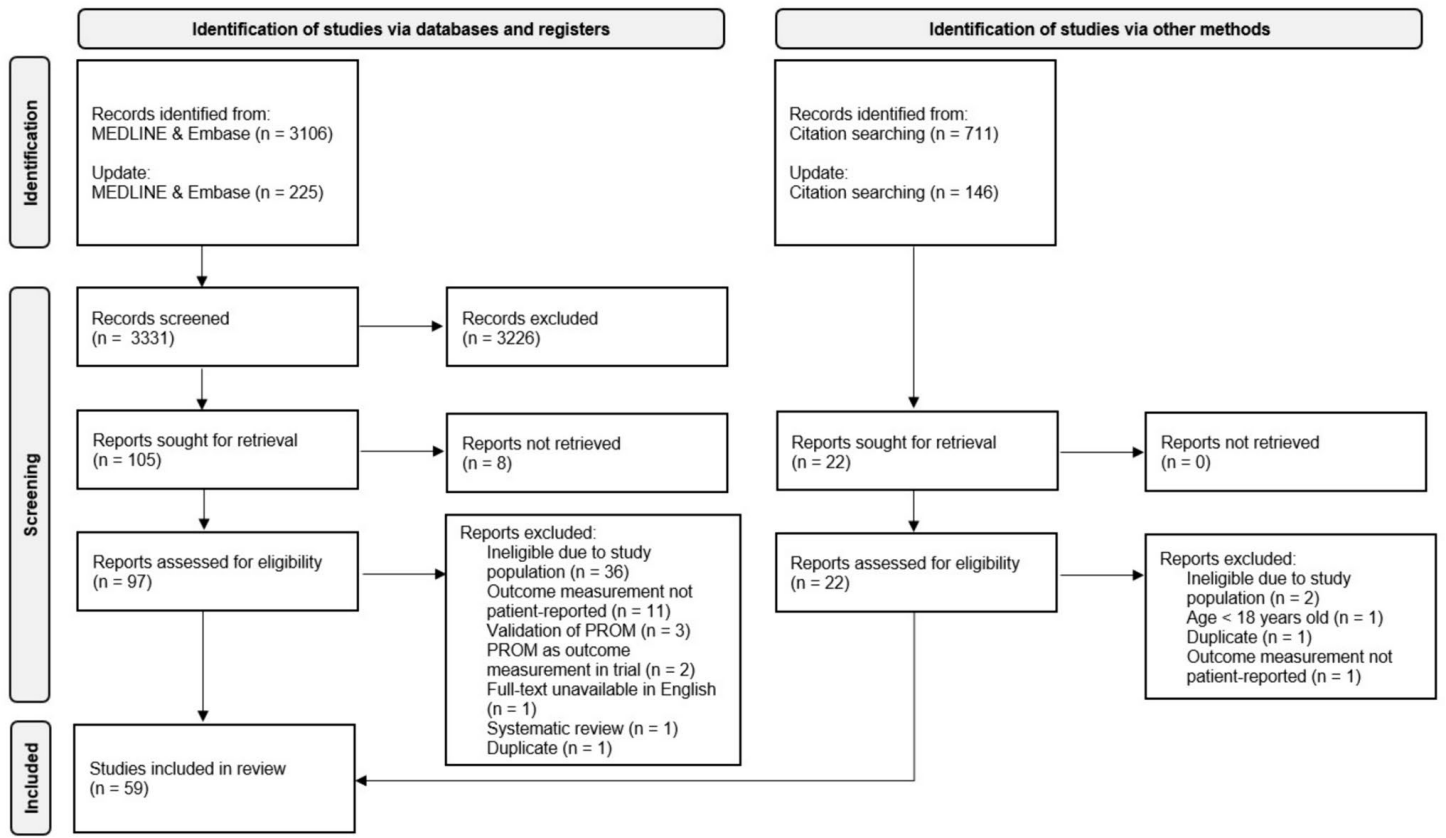
Flow diagram of search 1

**Fig. 3:**
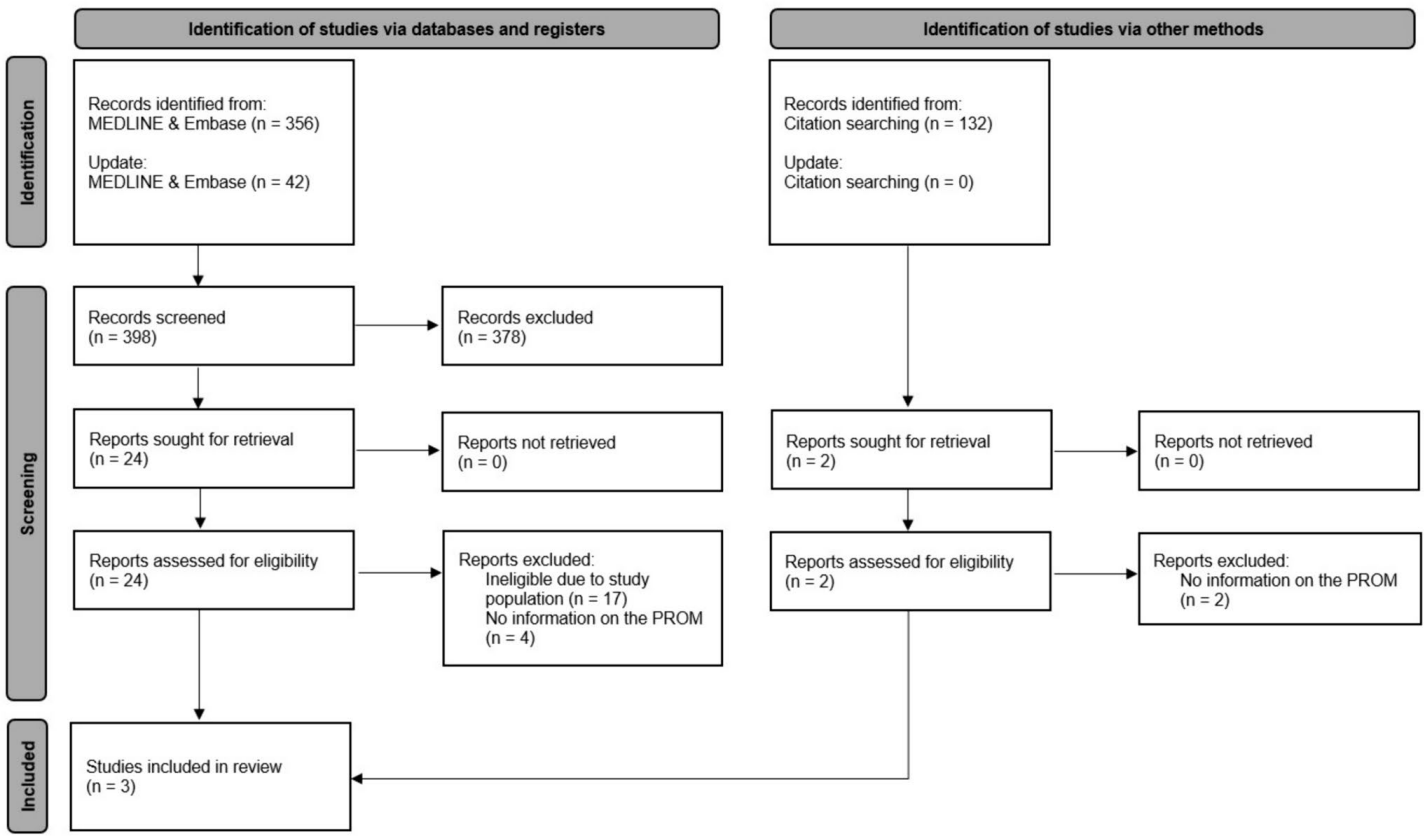
Flow diagram of search 2

Search 1, which aimed to identify all PROMs used in the STS population, produced 4,188 records. Tracking citations from available reviews on PROs in the sarcoma population [6–8, 27–35] led to 856 records. After screening, 127 reports were identified for retrieval, resulting in 119 reports assessed for eligibility. Fifty-nine studies were included in the review (refer to Fig. 2 for the flow diagram) [13–16, 18, 36–89]. Search 2 generated 530 records. Retrieval and assessment of 26 reports for eligibility resulted in three studies being included in the review (see Fig. 3 for the flow diagram) [54, 90, 91]. Supplementary Information 3 provides a list of all articles that underwent full-text review but were subsequently excluded, along with the reasons for their exclusion.

### Identification of patient-reported outcome measures

Following search 1, 59 studies were included in the review. Median year of publication 2018 [IQR 8]. Of these, 45 (76.3%) were cohort studies, 12 (20.3%) were cross-sectional studies, one (1.7%) was a (non-randomized) phase IV study and one (1.7%) was a cluster-randomized controlled trial (which presented longitudinal data without treatment randomization). Among cohort studies, 27 (45.8%) were prospective and 20 (33.9%) were retrospective. Monocenter studies accounted for 44 (74.6%) of the total, while multicenter studies compromised 14 (23.7%). The median sample size was 68 [IQR 105] patients, with a mean age of 57.0 (SD 7.0) years, and a median percentage of male patients at 52.4 [IQR 10.8] %. The overall quality varied across the studies: 12 (20.3%) were deemed poor, 39 (66.1%) were considered fair, and 8 (13.6%) were rated as good. Further details on study characteristics can be found in Supplementary Information 4 and Supplementary Information 5 provides information on risk of bias.

**Fig. 4:**
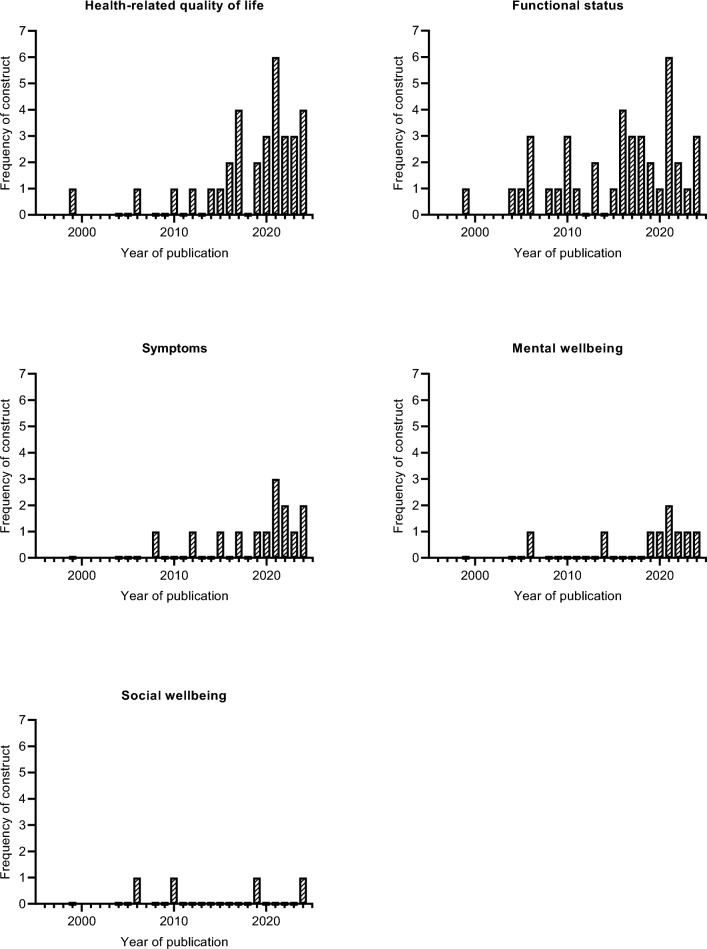
The frequency of constructs evaluated in the included studies per year of publication

Health-related quality of life was evaluated in 33 (55.9%) studies. In 40 (67.8%) studies, functional status was evaluated. Symptoms were the focus of evaluation in 14 (23.7%) studies, mental wellbeing in 9 (15.3%), and social wellbeing in 4 (6.8%) studies. Figure 4 illustrates the frequency of constructs evaluated in the included studies per year of publication. A total of 39 PROMs were used to assess PROs within the STS population; an overview of all PROMs is available in Supplementary Information 6. Among these, the TESS was the most commonly employed, utilized in 28 (47.5%) of the studies.

### Characteristics of patient-reported outcome measures

Table 2 illustrates the characteristics of the PROMs employed in the STS population. Among these, 7 (17.9%) PROMs focused on assessing health-related quality of life, 7 (17.9%) on functional status, 14 (35.9%) on symptoms, 9 (23.1%) on mental wellbeing, and 2 (5.1%) on social wellbeing. Of the total, 22 (56.4%) were generic PROMs, while 17 (43.6%) were disease-specific. Cancer patients were the target population in 10 (25.6%) measures, while sarcoma patients were the focus in 2 (5.1%) measures. These were the TESS and the Three-Item Cancer-Related Symptoms Questionnaire. Likert scaling was predominantly utilized as rating scale, accounting for 37 (94.9%) of all PROMs.

**Table 2:** Characteristics of patient-reported outcome measures

PROM (first reference)	Type	Construct(s)	Target population	Mode of administration	Items	Rating scale	Item score	Total score
*Health-related quality of life*								
EORTC-QLQ-C30 [97]	Disease-specific	Health-related quality of life (functional status, symptoms, global health, quality of life, financial impact)	Patients with cancer	Self-report	30	Item 1–7: no/yes, item 8–28: Likert scale 4-point, item 29–30: Likert scale 7-point	0–100	Score per scale (0–100)
SF-36 [98]	Generic	Health-related quality of life (physical functioning, role limitations because of physical health problems, bodily pain, social functioning, general mental health, role limitations because of emotional problems, vitality, general health perceptions)	Persons 14 years of age and older	Self-report or interview	36	Item 4–5: no/yes, other items: scale with Likert scale ranging from 3-point to 7-point	0–100	Scores determined by algorithm (T-score based on norm: general US population)
SF-8 [99]	Generic	Health-related quality of life (physical functioning, role limitations because of physical health problems, bodily pain, social functioning, general mental health, role limitations because of emotional problems, vitality, general health perceptions)	Persons 14 years of age and older	Self-report	8	Likert scale 5-point or 6-point	0–100	Scores determined by algorithm (T-score based on norm: general US population)
EQ-5D-3L [100]	Generic	Health-related quality of life (mobility, self-care, main activity, social relationships, pain, mood)	Adults	Self-report	5	3 levels and overall rating of health	Variable	Overall rate of health 0–100, score per level, summary index (based on a formula)
EQ-5D-5L [101]	Generic	Health (mobility, self-care, usual activities, pain/discomfort, anxiety/depression)	Adults	Self-report	5	5 levels and overall rating of health	Variable	Overall rate of health 0–100, score per level, summary index (based on a formula)
FACT-G [102]	Disease-specific	Health-related quality of life (physical, social, emotional and functional well-being)	Patients with cancer	Self-report	27	Likert scale 5-point	0–4	Weighted score per subscale, transformed to overall score of 0–108
PROMIS -Global Health [103]	Generic	Health (physical function, fatigue, pain, emotional distress, and social health)	Adults	Self-report	10	Likert scale 5-point	Variable	One score for physical health and one score for mental health
*Functional status*								
TESS [11]	Disease-specific	Functional status	Adult musculoskeletal oncology	Self-report	57 (28 UE and 29 LE)	Likert scale 5-point	Weighted	0–100
LEFS [104]	Disease-specific	Functional status	Patients with any LE musculoskeletal condition	Self-report	20	Likert scale 5-point	0–4	0–80
MHQ [105]	Disease-specific	Functional status	Patients with hand disorders	Self-report	37	Raw score per scale (in total 6 different scales), which is converted to a score 0–100	Weighted	0–100, the sum of each scale divided by 6
FAOS [106]	Disease-specific	Functional status	Patients with foot and ankle problems	Self-report	42	Likert scale 5-point, 5 subscales	0–4	Raw scores of the 5 subscales transformed to 0–100
SMFA [107]	Disease-specific	Functional status	Patients with musculoskeletal disease	Self-report	46	Two scales (34 and 12 items), Likert scale 5-point (1–5)	1–5	Sum of scales transformed by formula to 0–100
QuickDASH [108]	Disease-specific	Functional status and symptoms	Patients with any or multiple disorders of the UE	Self-report	11	Likert scale 5-point	1–5	Sum of responses transformed by formula to 0–100
PROMIS -Physical function [103]	Generic	Physical function	Adults	Self-report	Item bank 124; short form 10	Likert scale 5-point	Variable	T-score (score of 50 equals the mean of the US population)
*Symptoms*								
BPI-SF [109]	Generic	Pain	Adults	Self-report	9	Item 1: no/yes, item 2: body diagram, item 3–6 and 8–9: Likert scale 0–10, item 7: open-ended question	0–10	Per subscale (pain severity and pain interference): mean of Likert scale (0–10)
LENT-SOMA [110]	Disease-specific	Late toxicity of radiation therapy	Patients with cancer	Self-report or interview	37	Likert scale 4-point	1–4	None, all criteria are described separately
ISI [111]	Generic	Insomnia	Adults with insomnia complaints	Self-report	7	Likert scale 5-point	0–4	Summary of scores per item
MFI-20 [112]	Generic	Fatigue (general fatigue, physical fatigue, reduced activity, reduced motivation, mental fatigue)	Adults	Self-report	20	Likert scale 5-point	1–5	Score per scale (4–20)
NRS [113]	Generic	Pain	Adults	Self-report	1	Likert scale 11-point	0–10	0–10
MIDOS [114]	Disease-specific	Symptoms (pain, drowsiness, nausea, constipation, dyspnea, lymphedema, weakness, anxiety, well-being)	Palliative patients	Self-report	16	Likert scale 4-point	0–3	Summary of scores per item (0–48)
MDASI [115]	Disease-specific	Symptoms (severity and interference)	Patients with cancer	Self-report or interview	19	Likert scale 11-point	0–10	Per subscale (mean or summary of scores)
MSAS-SF [116]	Disease-specific	Symptoms (global distress, physical symptom distress, psychological symptom distress)	Patients with cancer	Self-report	32	Likert scale 5-point	0–4	Per subscale (mean: 0–4)
Three-item Cancer-Related Symptoms Questionnaire [17]	Disease-specific	Pain, cough, shortness of breath	Metastatic sarcomas	Self-report	3	Mild-moderate-severe (%)	None (%)	None
PROMIS—Pain Interference [103]	Generic	Pain	Adults	Self-report	Item bank 41; short form 6	Likert scale 5-point	Variable	T-score (score of 50 equals the mean of the US population)
PROMIS—Fatigue [103]	Generic	Fatigue	Adults	Self-report	Item bank 95; short form 7	Likert scale 5-point	Variable	T-score (score of 50 equals the mean of the US population)
PROMIS—Sleep disturbance [103]	Generic	Sleep	Adults	Self-report	Item bank 27; short form 8	Likert scale 5-point	Variable	T-score (score of 50 equals the mean of the US population)
FACIT-F [117]	Disease-specific	Fatigue	Patients with chronic (oncologic) illnesses	Self-report	13	Likert scale 5-point	0–4	Individual item score multiplied by 13, divided by number of items answered
PRO-CTCAE [118]	Disease-specific	Symptoms	Patients on cancer clinical trials	Self-report	124	Likert scale 5-point	0–4	None, score per attribute
*Mental wellbeing*								
HADS [119]	Generic	Anxiety, depression	Patients in a non-psychiatric hospital department	Self-report	14	Likert scale 4-point	0–3	Per subscale (anxiety/depression): summary of scores
CWS [120]	Disease-specific	Cancer worries	Patients with cancer	Self-report	8	Likert scale 4-point	1–4	8–32
IES [121]	Generic	Stress	Adults	Self-report	15 (7 intrusion and 8 avoidance items)	0 (not at all)—1 (rarely)—3 (sometimes)—5 (often)	0–1-3–5	Per subscale (intrusion: 0–35 and avoidance: 0–40), summary of scores (0–75)
NCCN Distress Thermometer [122]	Disease-specific	Distress	Patients with cancer	Self-report	1	Likert scale 11-point	0–10	0–10
WHO-5 [123]	Generic	Mental wellbeing	Children aged 9 and above	Self-report	5	Likert scale 6-point	0–5	0–25
PROMIS—Depression [103]	Generic	Depression	Adults	Self-report	Item bank 28; short form 8	Likert scale 5-point	Variable	T-score (score of 50 equals the mean of the US population)
PROMIS—Anxiety [103]	Generic	Anxiety	Adults	Self-report	Item bank 29; short form 7	Likert scale 5-point	Variable	T-score (score of 50 equals the mean of the US population)
WEMWBS [124]	Generic	Mental wellbeing	Adults	Self-report	14; short form 7	Likert scale 5-point	1–5	Summing the scores of each of the items
FoP-Q-SF^a^	Disease-specific	Fear of progression	Patients with cancer	Self-report	12	Likert scale 5-point	1–5	12–60
*Social wellbeing*								
RNL [125]	Generic	Participation (mobility, self-care, daily activity, recreational activity, family roles)	Adults after incapacitating illness or severe trauma	Self-report	11	Likert scale 10-point	1–10	Summary of scores (maximum 110), proportionally converted to a 100-point scale
PROMIS—Ability to participate [103]	Generic	Social function	Adults	Self-report	Item bank 14; short form 7	Likert scale 5-point	Variable	T-score (score of 50 equals the mean of the US population)

*US* United States of America, *UE* upper extremity, *LE* lower extremity, *PROM* patient-reported outcome measure

^a^Full-text of development study not available

### Methodological assessment of patient-reported outcome measures

After search 2, three studies were found that report on measurement properties of three PROMs in the STS population; the TESS, Quick Disability of the Arm, Shoulder and Hand (QuickDASH) and European Organization for Research and Treatment for Cancer Quality of Life Questionnaire (EORTC-QLQ-C30). Table 3 provides an overview of the characteristics of the included study populations. The sample sizes ranged from 14 to 136 patients, with ages spanning from 52 to 65 years old. Two of the studies included patients with localized STS disease, while one focused on advanced STS patients. All assessments were done in clinical settings. Language-wise, two studies were in Finnish and one in English. Response rates varied from 70 to 85%. In Table 4, we present the results of the measurement properties assessed against criteria for good measurement properties.

**Table 4:** Characteristics of the included study populations

PROM	References	Population			Disease		Instrument administration			Measurement properties
		n	Age^a^	Gender^b^	Stage	Treatment	Setting	Country (language)	Response rate (%)	
TESS	Kask et al. [90]	136	65.6 (14.4)	66 (48.5)	Local STS (lower extremity)	Surgery	Clinical	Finland (Finnish)	70	Structural validity, internal consistency, measurement invariance, reliability and hypotheses testing for construct validity
	Ketola et al. [91]	55	62.8 (14.6)	27 (49.1)	Local STS (upper extremity)	Limb-sparing surgery	Clinical	Finland (Finnish)	85	Internal consistency, measurement invariance and hypotheses testing for construct validity
QuickDASH	Ketola et al. [91]	55	62.8 (14.6)	27 (49.1)	Local STS (upper extremity)	Limb-sparing surgery	Clinical	Finland (Finnish)	85	Internal consistency, measurement invariance and hypotheses testing for construct validity
EORTC-QLQ-C30	Gough et al. [54]	27; 14^c^	52.2 (14)	21 (32)	Locally advanced, inoperable or metastatic STS	Palliative chemotherapy or under surveillance after first-line palliative chemotherapy having responded favorably	Clinical	United Kingdom (English)	84	Content validity (the relevance of the PROM items)

*PROM* patient-reported outcome measure, *STS* soft-tissue sarcoma

^a^Mean (SD) or median [IQR]

^b^Male patients (%)

^c^Twenty-seven patients were included for quantitative analysis; 14 patients for qualitative analysis

**Table 4:** Results of studies on measurement properties

PROM	Structural validity			Internal consistency		Measurement invariance		Reliability		Hypotheses testing for construct validity	
	n	MQ	Result (rating)	MQ	Result (rating)	MQ	Result (rating)	MQ	Result (rating)	MQ	Result (rating)
TESS [90]	136	Inadequate	EFA: factor 1 explaining 74.4% of the variance; factor 2 6.4%; factor 3 4.2% and factor 4 3.3% (?)	Very good	Cronbach’s alpha 0.97 (95% CI = 0.97–0.98) (?/+)	Inadequate	Significant correlation of TESS with age (rho = − 0.23 and p = 0.006) and BMI (rho = − 0.25 and p = 0.006). No difference in gender (t test, p = 0.143) (−)	Doubtful	ICC 0.95 (95% CI = 0.93–0.96 and p < 0.001) (+)	Inadequate	Results in line with 8 hypotheses Results not in line with 2 hypotheses (+)
TESS [91]	55			Very good	Cronbach’s alpha 0.97 (?/+)	Inadequate	No difference in BMI and age, Spearman’s correlation − 0.17 for age and − 0.09 for BMI. Sex insignificant (t test, p = 0.53). (+)			Inadequate	Results in line with 8 hypotheses Results not in line with 2 hypotheses (+)
QuickDASH [91]	55			Inadequate	Cronbach’s alpha 0.930 (?/+)	Inadequate	No difference in BMI and age, Spearman’s correlation 0.03 for age and 0.01 for BMI. Gender insignificant (t test, p = 0.92). (+)			Inadequate	Results in line with 8 hypotheses Results not in line with 2 hypotheses (+)

*PROM* patient-reported outcome measure, *MQ* methodological quality, *EFA* exploratory factor analysis, *BMI* body mass index, *ICC* intraclass correlation coefficient

#### Content validity

The TESS was the sole PROM in the review developed for STS patients, yet it was rated inadequate according to the COSMIN guidelines. The development study of the Three-item Cancer-Related Symptoms Questionnaire, which potentially involved STS patients, was unavailable. One study was performed to evaluate content validity of a PROM in the STS population. The relevance of the PROM items of the EORTC-QLQ-C30 were evaluated by asking patients what constitutes health-related quality of life. The overall rating of the quality of the content validity study was inadequate. From the qualitative interviews, eight factors were described as relevant for a good health-related quality of life in advanced STS patients. These factors were being free from pain/symptoms, time with family and friends, help with anxiety, loss of independence/control over life, enjoyment of job, outdoor activities, holidays and financial stability. Of these eight factors, three were not mentioned in the EORTC-QLQ-C30. These factors are loss of independence/control over life, enjoyment of job and holidays.

#### Internal structure

In evaluating the TESS, structural validity was examined in 136 patients, with internal consistency and measurement invariance studied in 191 patients. The analysis of structural validity revealed a high risk of bias. An exploratory factor analysis was conducted with one factor explaining 74.4% of the variance of all items. Internal consistency showed low risk of bias, with Cronbach’s alpha 0.97 (95% CI 0.97–0.98). Results for measurement invariance were inconsistent, possibly due to variations in study populations. Risk of bias for the assessment of measurement invariance was high and one study reports a significant correlation of TESS with age (rho = − 0.23, p = 0.006) and BMI (rho = − 0.25, p = 0.006). As for the QuickDASH, internal consistency and measurement invariance were assessed in 55 patients, with a high risk of bias. Cronbach’s alpha was 0.930 (no CI reported) and there were no significant correlations (with BMI, age, gender).

#### Other measurement properties

Reliability of the TESS was evaluated in 136 patients and construct validity in 191 patients. Risk of bias for reliability was moderate, with an ICC of 0.95 (95% CI 0.93–0.96, p < 0.001). Risk of bias for the assessment of construct validity was high and results were in accordance with hypotheses. For the QuickDASH construct validity was assessed in 55 patients, with high risk of bias. The results were in line with hypotheses.

#### Interpretability and feasibility

There was no floor effect for the TESS and QuickDASH. However, a ceiling effect was observed for both, with 27% of study participants achieving the maximum score on the TESS and 20% on the QuickDASH.

#### Recommendation

Due to the limited availability of evidence, a high risk of bias, and inconsistencies in findings, we were unable to pool or summarize the results. None of the PROMs utilized in the STS population can be recommended for use based on the current evidence and corresponding COSMIN analyses.

## Discussion

This systematic review presents an overview of all PROMs utilized in the STS population and a methodological evaluation of these PROMs. Functional status continues to be the predominantly researched construct, highlighting gaps in knowledge related to mental and social wellbeing. Thirty-nine different PROMs were identified, with the TESS, EORTC-QLQ-C30 and EQ-5D-3L being the most frequently utilized in the STS population. Given the scarcity of evidence, high risk of bias, and inconsistencies in results, it is not feasible to recommend any of the PROMs for use in the STS population at this time. Notably, there persists an inadequacy of knowledge on content validity of PROMs utilized in the STS population, the most important measurement property. The development of only one PROM, the TESS, involved STS patients and one content validity study has been conducted. Both were of inadequate quality; the content validity study additionally revealed insufficient content validity of the EORTC-QLQ-C30 in the STS population.

Research conducted with PROMs of questionable or undetermined quality is prevalent [92], which is consistent with the review’s findings. This could be attributed to the fact that more than half of the employed PROMs were generic. Generic PROMs, such as the EQ-5D, have proven to result in sufficient measurement properties in wide variations of populations [90, 93], so validation in specific populations is sometimes argued to be unnecessary. Measurement properties of generic PROMs can however vary, for instance the measurement properties of the EQ-5D in patients with mental health disorders were found to be doubtful [93, 94]. Hence, validation of generic PROMs in specific populations is recommended before use, which is in line with the COSMIN methodology [21].

Pressure on health care systems to improve quality and control costs has resulted in the development of value-based care [95]. In a value-based health care system, efficiency is analyzed by calculating quality-adjusted life years (QALYs). The QALY is a measure of survival weighted by a coefficient that expresses a state of health (utility value) in comparison with perfect health [91]. PROMs, such as the EQ-5D, are used in value-based care to determine the patients’ health state and corresponding utility value. The utility value is therefore directly related to the ability of the PROM to measure outcome of a health state in a specific patient population. Assessing PROM quality is ethically important, as patient invest time and effort in providing information about their health status. Also, the relevance of evaluating PROMs lies in producing credible and generalizable data to ensure evidence-based medicine, and promotes individualized and value-based healthcare.

Recent reviews have emphasized the importance of developing a specific instrument to capture the patient experience of STS diagnosis, treatment and follow-up [28, 30–32]. The rationale for developing a new instrument is to analyze the specific experience of diagnosis, treatment and follow-up of STSs, as its rarity is expected to result in a different experience than tumors of more frequent occurrence. The TESS, a sarcoma-specific PROM, has been the PROM used most frequently in the STS population [11]. Based on our analysis, there is limited supporting evidence for the TESS effectively assessing functional status. Since the development of the TESS, improved treatment options have resulted in decreased morbidity. The qualitative study of Martins et al. [96] including STS patients at various stages from diagnosis indicates that 68% of issues affecting STS patients were related to mental wellbeing, such as anxiety, depression and distress. In that study, it is stated that their top-rated items on functional status do not reflect those included in existing measures. The construct and items evaluated by the TESS may therefore not be that relevant in the current time. These findings, along with the prospective outlook on value-based health care, stress the importance of systematically analyzing measurement properties of existing PROMs, as well as performing qualitative research in specific patient populations to determine relevant content.

Considerable effort has been put into identifying all PROMs utilized in the STS population. This involved thorough searches of two large biomedical databases without time limits, as well as citation tracking. Some PROMs that could be applicable for use in STS patients may not have been included because they have not been utilized to measure outcomes in the STS population. Additionally, the search was limited to two biomedical databases and may have missed articles in other fields. No grey literature review was undertaken. The current review is the first to explore methodological quality of PROMs in the STS population using the COSMIN guidelines. Owing to the paucity of available evidence, we could not offer summarized or pooled results. Nevertheless, this review marks an initial move towards elevating the standard of research with PROMs in the STS population. Consolidating the findings of the searches pertaining to our two objectives within a unified review facilitates access to information on previous employed PROMs and our recommendations from existent evidence.

To identify PROMs, we opted to exclude trials that randomize treatment. While this may be viewed as a limitation of our review, it serves to mitigate bias in our findings. By focusing solely on PROMs which share a common intended purpose, we ensure greater consistency and reliability in our analysis. The review is limited by the absence of protocol registration prior to commencement, a step that was overlooked due to the lack of anticipation for duplication. However, our eligibility criteria, design, and objectives remained consistent throughout the process. The assessment of content validity was tailored to fit the target population of the current review and the limited available literature in the context. According to the COSMIN guidelines, it is recommended to assess all PROM development studies based on their target population. We focused solely on evaluating PROMs developed involving STS patients, as this aligns with the specific target population of our review. In addition, reviewers are recommended to rate the content validity of the PROMs, considering evidence from PROM development studies and content validity studies within the specific target population. The scarcity of knowledge on the content of (aspects of) health-related quality of life in STS patients and the absence of both PROM development and content validity studies posed limitations. These limitations prevented reviewers from carrying out the assessment, as it would lack an evidence-based foundation.

The utilization of PROMs has seen a rise in the last twenty-five years, leading to a substantial increase in the number of available PROMs. This review marks the first systematic exploration of evidence regarding the measurement properties of PROMs used in the STS population. Given the restricted available evidence on the quality of PROMs employed in the STS population and considering future perspectives, now is an opportune time to change the narrative. This involves exploring relevant content specific to the STS population and subsequently choosing the most appropriate PROM to measure it. While existing PROMs may have potential suitability for application in the STS population, it is imperative to investigate their methodological quality to ensure the validity and reliability of outcomes.

## Supplementary Information

Below is the link to the electronic supplementary material.

Supplementary file1 (DOCX 31 KB)

Supplementary file2 (DOCX 17 KB)

Supplementary file3 (XLSX 28 KB)

Supplementary file4 (DOCX 116 KB)

Supplementary file5 (XLSX 13 KB)

Supplementary file6 (DOCX 16 KB)

Supplementary file1 (DOCX 16 KB)

## Data Availability

All data are publicly available and can be found either within the tables of the review or in the Supplementary Information.
