# Supplementary material for: Twenty-five years of experience with patient-reported outcome measures in soft-tissue sarcoma patients: a systematic review
Source: Qual Life Res. 2024 Sep 11;33(12):3189–211. doi: 10.1007/s11136-024-03755-4 (PMC11599342; doi:10.1007/s11136-024-03755-4)
Supplement: Supplementary file 2 — Supplementary file2 (DOCX 17 KB) [file 11136_2024_3755_MOESM2_ESM.docx]

**Supplementary Information 2**. Search strategies Embase

**Search strategy 1: Identification of PROMs**

('sarcoma'/exp OR sarcoma*:ab,ti,kw OR ‘musculoskeletal tumo*’:ab,ti,kw)

AND

('patient-reported outcome'/exp OR ‘patient reported’:ab,ti,kw OR promis:ab,ti,kw OR 'mental health'/exp OR 'anxiety'/exp OR 'depression'/exp OR ‘mental health’:ab,ti,kw OR 'health status'/exp OR ‘health status’:ab,ti,kw OR anxiet*:ab,ti,kw OR depress*:ab,ti,kw OR ‘social health’:ab,ti,kw OR psychosocial*:ab,ti,kw OR physical*:ab,ti,kw OR participat*:ab,ti,kw OR wellbeing:ab,ti,kw OR 'morbidity'/exp OR morbidit*:ab,ti,kw OR 'quality of life'/exp OR ‘life quality’:ab,ti,kw OR qol:ab,ti,kw OR hrqol:ab,ti,kw OR questionnaire*:ab,ti,kw) AND ('cohort analysis'/exp OR cohort:ab,ti,kw OR ‘follow-up’:ab,ti,kw OR prospective:ab,ti,kw OR observational:ab,ti,kw)

NOT

(('child'/exp OR 'adolescent'/exp OR 'infant'/exp) NOT 'adult'/exp) NOT (‘bone sarcoma’:ti OR 'osteosarcoma'/exp) NOT 'conference abstract'/it

**Search strategy 2: Methodological evaluation of PROMs**

('sarcoma'/exp OR sarcoma*:ab,ti,kw OR ‘musculoskeletal tumo*’:ab,ti,kw)

AND

((‘Toronto extremity salvage score*’ OR tess* OR ‘EORTC-QLQ-C30’ OR ‘EORTC quality of life questionnaire’ OR ‘EQ-5D-3L’ OR ‘Euro Quality of Life 5 Dimensions 3 Levels’ OR ‘Short Form Health Survey’ OR ‘SF-36’ OR ‘Lower Extremity Functional Scale*’ OR LEFS OR ‘Short Form 8’ OR ‘SF-8’ OR ‘Hospital Anxiety and Depression Scale*’ OR HADS OR ‘patient reported outcome measurement information system’ OR ‘Disabilities of the Arm, Shoulder and Hand*’ OR QuickDASH* OR ‘Brief Pain Inventory Short Form*’ OR ‘BPI-SF’ OR ‘Re-integration to Normal Living*’ OR RNL OR ‘EuroQol five-dimension’ OR ‘EQ-5D-5L’ OR ‘Michigan Hand Outcomes Survey*’ OR MHQ OR ‘Foot and Ankle Outcomes Survey*’ OR FAOS OR PROMIS OR ‘Patient-Reported Outcome Measurement System*’ OR ‘Late Effects Normal Tissues-Subjective, Objective, Management, Analytic’ OR ‘LENT-SOMA’ OR ‘Short Musculoskeletal Function Assessment*’ OR SMFA OR ‘Cancer Worry Scale*’ OR CWS OR ‘World Health Organization Five Well being Index*’ OR ‘WHO-5’ OR ‘Insomnia Severity Index*’ OR ISI OR ‘Multidimensional Fatigue Inventor*’ OR ‘MFI-20’ OR ‘Impact of Event Scale*’ OR IES OR ‘Numeric Rating Scale*’ OR NRS OR ‘NCCN Distress Thermometer’ OR ‘National Comprehensive Cancer Network Distress Thermometer’ OR ‘Minimal Documentation System*’ OR MIDOS OR MDASI OR MSAS SF OR ‘Three item Cancer Related Symptoms Questionnaire*’ OR ‘Functional Assessment of Cancer Therapy’ OR ‘FACT G’ OR ‘MD Anderson Symptom Inventory’ OR ‘Memorial Symptom Assessment Scale Short Form’ OR 'functional assessment of chronic illness therapy – fatigue' OR 'facit-f' OR 'facit-fatigue' OR 'functional assessment of chronic illness therapy fatigue scale' OR 'pro ctcae' OR 'patient-reported outcomes version of the common terminology*' OR 'warwick-edinburgh mental well-being scale' OR 'fop q sf' OR 'fear of progression questionnaire short form'):ab,ti,kw)

AND

(‘intermethod comparison’/exp OR ‘data collection method’/exp OR ‘validation study’/exp OR

‘feasibility study’/exp OR ‘pilot study’/exp OR ‘psychometry’/exp OR ‘reproducibility’/exp OR

reproducib*:ab,ti OR ‘audit’:ab,ti OR psychometr*:ab,ti OR clinimetr*:ab,ti OR clinometr*:ab,ti OR

‘observer variation’/exp OR ‘observer variation’:ab,ti OR ‘discriminant analysis’/exp OR ‘validity’/exp

OR reliab*:ab,ti OR valid*:ab,ti OR ‘coefficient’:ab,ti OR ‘internal consistency’:ab,ti OR

(cronbach*:ab,ti AND (‘alpha’:ab,ti OR ‘alphas’:ab,ti)) OR ‘item correlation’:ab,ti OR ‘item

correlations’:ab,ti OR ‘item selection’:ab,ti OR ‘item selections’:ab,ti OR ‘item reduction’:ab,ti OR

‘item reductions’:ab,ti OR ‘agreement’:ab,ti OR ‘precision’:ab,ti OR ‘imprecision’:ab,ti OR ‘precise

values’:ab,ti OR ‘test-retest’:ab,ti OR (‘test’:ab,ti AND ‘retest’:ab,ti) OR (reliab*:ab,ti AND (‘test’:ab,ti

OR ‘retest’:ab,ti)) OR ‘stability’:ab,ti OR ‘interrater’:ab,ti OR ‘inter-rater’:ab,ti OR ‘intrarater’:ab,ti OR

‘intra-rater’:ab,ti OR ‘intertester’:ab,ti OR ‘inter-tester’:ab,ti OR ‘intratester’:ab,ti OR ‘intratester’:ab,ti

OR ‘interobeserver’:ab,ti OR ‘inter-observer’:ab,ti OR ‘intraobserver’:ab,ti OR ‘intraobserver’:ab,ti

OR ‘intertechnician’:ab,ti OR ‘inter-technician’:ab,ti OR ‘intratechnician’:ab,ti OR ‘intratechnician’:ab,ti

OR ‘interexaminer’:ab,ti OR ‘inter-examiner’:ab,ti OR ‘intraexaminer’:ab,ti OR ‘intraexaminer’:ab,ti

OR ‘interassay’:ab,ti OR ‘inter-assay’:ab,ti OR ‘intraassay’:ab,ti OR ‘intra-assay’:ab,ti

OR ‘interindividual’:ab,ti OR ‘inter-individual’:ab,ti OR ‘intraindividual’:ab,ti OR ‘intra-individual’:ab,ti

OR ‘interparticipant’:ab,ti OR ‘inter-participant’:ab,ti OR ‘intraparticipant’:ab,ti OR ‘intraparticipant’:ab,ti

OR ‘kappa’:ab,ti OR ‘kappas’:ab,ti OR ‘coefficient of variation’:ab,ti OR

repeatab*:ab,ti OR (replicab*:ab,ti OR ‘repeated’:ab,ti AND (‘measure’:ab,ti OR ‘measures’:ab,ti OR

‘findings’:ab,ti OR ‘result’:ab,ti OR ‘results’:ab,ti OR ‘test’:ab,ti OR ‘tests’:ab,ti)) OR generaliza*:ab,ti

OR generalisa*:ab,ti OR ‘concordance’:ab,ti OR (‘intraclass’:ab,ti AND correlation*:ab,ti) OR

‘discriminative’:ab,ti OR ‘known group’:ab,ti OR ‘factor analysis’:ab,ti OR ‘factor analyses’:ab,ti OR

‘factor structure’:ab,ti OR ‘factor structures’:ab,ti OR ‘dimensionality’:ab,ti OR subscale*:ab,ti OR

‘multitrait scaling analysis’:ab,ti OR ‘multitrait scaling analyses’:ab,ti OR ‘item discriminant’:ab,ti OR

‘interscale correlation’:ab,ti OR ‘interscale correlations’:ab,ti OR (‘error’:ab,ti OR ‘errors’:ab,ti AND

(measure*:ab,ti OR correlat*:ab,ti OR evaluat*:ab,ti OR ‘accuracy’:ab,ti OR ‘accurate’:ab,ti OR

‘precision’:ab,ti OR ‘mean’:ab,ti)) OR ‘individual variability’:ab,ti OR ‘interval variability’:ab,ti OR ‘rate

variability’:ab,ti OR ‘variability analysis’:ab,ti OR (‘uncertainty’:ab,ti AND (‘measurement’:ab,ti OR

‘measuring’:ab,ti)) OR ‘standard error of measurement’:ab,ti OR sensitiv*:ab,ti OR responsive*:ab,ti

OR (‘limit’:ab,ti AND ‘detection’:ab,ti) OR ‘minimal detectable concentration’:ab,ti OR

interpretab*:ab,ti OR (small*:ab,ti AND (‘real’:ab,ti OR ‘detectable’:ab,ti) AND (‘change’:ab,ti OR

‘difference’:ab,ti)) OR ‘meaningful change’:ab,ti OR ‘minimal important change’:ab,ti OR ‘minimal

important difference’:ab,ti OR ‘minimally important change’:ab,ti OR ‘minimally important

difference’:ab,ti OR ‘minimal detectable change’:ab,ti OR ‘minimal detectable difference’:ab,ti OR

‘minimally detectable change’:ab,ti OR ‘minimally detectable difference’:ab,ti OR ‘minimal real

change’:ab,ti OR ‘minimal real difference’:ab,ti OR ‘minimally real change’:ab,ti OR ‘minimally real

difference’:ab,ti OR ‘ceiling effect’:ab,ti OR ‘floor effect’:ab,ti OR ‘item response model’:ab,ti OR

‘irt’:ab,ti OR ‘rasch’:ab,ti OR ‘differential item functioning’:ab,ti OR ‘dif’:ab,ti OR ‘computer adaptive

testing’:ab,ti OR ‘item bank’:ab,ti OR ‘cross-cultural equivalence’:ab,ti)
