# Supplementary material for: Twenty-five years of experience with patient-reported outcome measures in soft-tissue sarcoma patients: a systematic review
Source: Qual Life Res. 2024 Sep 11;33(12):3189–211. doi: 10.1007/s11136-024-03755-4 (PMC11599342; doi:10.1007/s11136-024-03755-4)
Supplement: Supplementary file 4 — Supplementary file4 (DOCX 116 KB) [file 11136_2024_3755_MOESM4_ESM.docx]

**Supplementary Information 4.** Study characteristics (of the included studies following the first search)

| **Authors** | **Year** | **Study type** | **Study design** | **Inclusion period** | **Population** | **Patients (STS cohort)** | **Age (median)** | **Sex (% male)** | **Primary endpoint** | **PROM** | **Timing of evaluation** |
| --- | --- | --- | --- | --- | --- | --- | --- | --- | --- | --- | --- |
| Ahmed et al. [1] | 2020 | Monocenter, retrospective | Cohort | 1991-2005 | Hand/foot sarcoma | 54 | 40.2 | 52 | Functional outcome | MHQ, TESS-UE, FAOS, TESS-LE | Long-term follow-up (median time from surgery to survey 15.7 years) |
| Andersen et al. [2] | 2024 | Monocenter | Cross-sectional | 2004-2021 | STS, primary tumor in an extremity, at least one year of follow-up data available | 331 | 56 | 55.6 | The extent to which biological function, symptoms, and functional status are associated with general health perceptions | FACIT-F, EQ-5D-3L, TESS, RNL, EQ-VAS | 12-month postoperative evaluation |
| Baysal et al. [3] | 2021 | Monocenter, retrospective | Cohort | 2012-2018 | UE sarcoma | 32 | 53 | 65.9 | Survival | SF-36, QuickDASH | Long-term follow-up (median time from surgery to survey 33 months) |
| Brandes et al. [4] | 2021 | Monocenter, retrospective | Cohort | 2012-2018 | Advanced STS | 34 | 59 | 54 | Patterns of symptoms in STS patients who have been treated in a hospital-based palliative setting | NRS, NCCN distress thermometer, MIDOS | At admission and discharge to the palliative care unit |
| **Authors** | **Year** | **Study type** | **Study design** | **Inclusion period** | **Population** | **Patients (STS cohort)** | **Age (median)** | **Sex (% male)** | **Primary endpoint** | **PROM** | **Timing of evaluation** |
| Callegaro et al. [5] | 2015 | Monocenter | Cross-sectional | 2002-2011 | RPS | 95 | 55 | 46.3 | Long-term morbidity (functional outcome, pain, renal failure) | BPI-SF, LEFS | Long-term follow-up (median time from surgery to survey 49 months) |
| Cassidy et al. [6] | 2016 | Monocenter, retrospective | Cohort | 1977-2010 | Hand/foot sarcoma | 11 | 49 | NR | Local control, survival, risk of treatment-related toxicity, functional outcome | TESS | NR |
| Cribb et al. [7] | 2010 | Monocenter, retrospective | Cohort | 1992-2007 | STS of foot/ankle | 27 | 47 | 44.4 | Treatment and functional outcome | TESS | NR |
| Dalton et al. [8] | 2022 | Monocenter, retrospective | Cohort | 7-2015 until10-2019 | STS | 127 | 60.5 | 62 | Perioperative changes and outcomes for patients undergoing resection of benign versus malignant STS | PROMIS physical function, pain interference, depression | At 2 and 6 weeks postoperatively |
| **Authors** | **Year** | **Study type** | **Study design** | **Inclusion period** | **Population** | **Patients (STS cohort)** | **Age (median)** | **Sex (% male)** | **Primary endpoint** | **PROM** | **Timing of evaluation** |
| Davidge et al. [9] | 2010 | Multicenter, retrospective | Cohort | 2001-2007 | ESTS | 247 | NA | NA | Postoperative function and health-status in ESTS patients undergoing limb salvage with and without soft-tissue reconstruction | TESS, RNL, EQ-5D-5L (EQ-5D-VAS) | Postoperative (1-2 years following surgery) |
| Davidson et al. [10] | 2016 | Monocenter, prospective | Cohort | 2001-2009 | STS | 220 | 54.4 | 59 | Change in health-related quality of life at diagnosis and at 1 year following surgery | EQ-5D-3L, TESS | Preoperative and 1 year postoperatively |
| Davis et al. [11] | 1999 | Monocenter, prospective | Cohort | 1986-1995 | LE STS | 80 | 51 | 51.2 | Tumor and treatment variables predictive of functional outcome | TESS, SF-36 | Long-term follow-up (mean time from surgery to functional assessment 45 months) |
| Drabbe et al. [12] | 2021 | Multicenter | Cross-sectional | NA | Sarcoma | 1099 | 62 | 54.2 | Health-related quality of life | EORTC-QLQ-C30 | NA |
| Eichler et al. [13] | 2020 | Multicenter | Cross-sectional | NA | Sarcoma | 1113 | 52.6 | 51.3 | Health-related quality of life | EORTC-QLQ-C30 | NA |
| **Authors** | **Year** | **Study type** | **Study design** | **Inclusion period** | **Population** | **Patients (STS cohort)** | **Age (median)** | **Sex (% male)** | **Primary endpoint** | **PROM** | **Timing of evaluation** |
| Fiore et al. [14] | 2021 | Monocenter, prospective | Cohort | 2014-2016 | RPS | 58 | 62.5 | 60.3 | Clinical outcomes (morbidity, renal function) and PROMs (quality of life, lower limb function, pain intensity) | EORTC-QLQ-C30, LEFS, BPI-SF | Day before surgery, 4 months after surgery and 1 year after surgery |
| Fischer et al. [15] | 2015 | Monocenter, retrospective | Cohort | 1992-2002 | STS of the thigh | 43 | 59.2 | 39.5 | Outcome of hamstring transfer for quadriceps reconstruction after massive tumor resection from the anterior thigh | SF-36 | NR |
| Gerrand et al. [16] | 2004 | Monocenter, prospective | Cohort | 4-1994 until 3-1999 | LE STS | 207 | 54 | 48.8 | The influence of anatomical location on functional scores | TESS | During follow-up of minimum 1 year following surgery |
| Ghert et al. [17] | 2005 | Monocenter, prospective | Cohort | 1989-2000 | STS with vascular reconstruction | 19 | 48.3 | 63 | Surgical, oncological and functional outcomes | TESS | Preoperative and postoperative at 1 year following surgery |
| **Authors** | **Year** | **Study type** | **Study design** | **Inclusion period** | **Population** | **Patients (STS cohort)** | **Age (median)** | **Sex (% male)** | **Primary endpoint** | **PROM** | **Timing of evaluation** |
| Götzl et al. [18] | 2019 | Monocenter | Cross-sectional | 2004-2014 | ESTS | 182 | 58 | NR | Health-related quality of life in extremity STS patients treated with and without RT | EORTC-QLQ-C30 | Time between treatment and questionnaire 65 months |
| Gough et al. [19] | 2017 | Monocenter | Cross-sectional | 3-2011 until 9-2012 | Metastatic or locally advanced inoperable STS | 113 | 59 | 32.7 | Symptom prevalence and severity in advanced STS patients undergoing palliative treatment | MSAS-SF | NA |
| Gough et al. [20] | 2019 | Monocenter, prospective | Cohort | 2011-2012 | Advanced STS | 66 | 55 | 32 | The individual constituents of HRQoL in patients undergoing first line palliative chemotherapy and active surveillance (after successful completion of first line chemotherapy) | EORTC-QLQ-C30 | At 2, 6, 12 and 18 weeks after commencing chemotherapy or active surveillance |
| **Authors** | **Year** | **Study type** | **Study design** | **Inclusion period** | **Population** | **Patients (STS cohort)** | **Age (median)** | **Sex (% male)** | **Primary endpoint** | **PROM** | **Timing of evaluation** |
| Heaver et al. [21] | 2016 | Monocenter, prospective | Cohort | 2010-2015 | Sarcoma | 198 | NR | NR | To identify what patient factors affect outcome after surgery | TESS | Every follow-up appointment (mean follow up UE: 29.1 months, mean follow-up LE: 26 months) |
| Homsy et al. [22] | 2022 | Monocenter, prospective | Cohort | 2014-2020 | STS patients requiring major vessel reconstruction in conjunction with tumor resection in the proximal thigh | 6 | 75 | 59 | Early and long-term outcome | TESS | Clinically |
| Hoven-Gondrie et al. [23] | 2008 | Monocenter, prospective | Cohort | 1991-2003 | Locally advanced STS | 32 | 47 | 44 | Long-term toxicity after ILP | LENT-SOMA | Long-term follow-up (median 88 months) |
| Jakob et al. [24] | 2022 | Multicenter, prospective | Cohort | 9-2017 until 2-2019 | RPS patients (M0) | 40 | 54 | 47 | Patient, tumor and treatment characteristics of the PROSa-RPS cohort in comparison to the TARPS-WG cohort | EORTC QLQ-C30 | During visits |
| Jones et al. [25] | 2010 | Monocenter, prospective | Cohort | NR | STS of the thigh, groin, pelvis | 10 | 64.7 | 0 | The functional implications for patients following femoral nerve resection | TESS | Follow-up (mean 46 months) |
| **Authors** | **Year** | **Study type** | **Study design** | **Inclusion period** | **Population** | **Patients (STS cohort)** | **Age (median)** | **Sex (% male)** | **Primary endpoint** | **PROM** | **Timing of evaluation** |
| Kapoor et al. [26] | 2018 | Monocenter, retrospective | Cohort | 1990-2017 | UE and LE STS | 178 | NR | NR | Overall clinical outcomes of limb reconstruction following surgery | QuickDASH, LEFS | Postoperative |
| Kasper et al. [27] | 2024 | Multicenter, prospective | Non-interventional phase IV study | 2-2-2017 until 31-12-2021 | Advanced or metastatic STS of intermediate or high grade | 69 | 78 | 56.5 | Overall survival, quality of life and individual clinical benefit | EORTC-QLQ-c30, PRO-CTCAE | Baseline, during treatment, end of treatment and 3- to 6-month follow-up |
| Kemp et al. [28] | 2011 | Monocenter, retrospective | Cohort | 1997-2006 | Low-grade liposarcomas of the forearm | 8 | 61 | 75 | Functional and oncological outcome following planned marginal excision of low-grade liposarcomas of the forearm with nerve involvement | TESS | After surgery (mean 23 months) |
| Kokkali et al. [29] | 2022 | Multicenter, prospective | Cohort | 21-12-2015 until 6-6-2018 | Locally advanced or advanced STS patients treated by trabectedin | 52 | 58.3 | 37.5 | Progression free survival at 6 months post-treatment initiation | G-MDASI, EQ-5D-3L | At enrollment, 6 weeks and 12 weeks |
| Komatsu et al. [30] | 2021 | Monocenter, prospective | Cohort | 2011-2017 | Inoperable bone sarcoma and STS | 24 | NR | NR | Quality of life, functional outcome | SF-8 | At 1, 3, 6, 12 and 24 months after treatment |
| **Authors** | **Year** | **Study type** | **Study design** | **Inclusion period** | **Population** | **Patients (STS cohort)** | **Age (median)** | **Sex (% male)** | **Primary endpoint** | **PROM** | **Timing of evaluation** |
| Kruiswijk et al. [31] | 2024 | Multicenter, prospective | Cluster-randomized controlled trial (the use of a clinical prediction model during clinical decision making) | 8-2021 until 12-2023 | High-grade extremity STS treated with curative intent | 97 | 64 | 50.0 | Changes in health-related quality of life from time of diagnosis until one year thereafter of a homogeneous high grade extremity sarcoma population, separately addressing adult and elderly (>65 years) patients | PROMIS Global Health, PROMIS Physical Function, EQ-5D-5L | Time of diagnosis, one week, three months, 6 months and 12 months after treatment decision |
| Lim et al. [32] | 2020 | Monocenter | Cross-sectional | 1999-2018 | RPS in Asian patients | 32 | 59 | 53.1 | Quality of life | EORTC-QLQ-C30 | 56,3% of patients < 2 years post-surgery, the remaining > 2 years post-surgery |
| MacArthur et al. [33] | 2019 | Monocenter, retrospective | Cohort | 2009-2013 | LE STS | 17 | 53 | NR | Functional outcome following lower extremity soft-tissue resection and reconstruction | TESS | At least 1 year post reconstruction |
| **Authors** | **Year** | **Study type** | **Study design** | **Inclusion period** | **Population** | **Patients (STS cohort)** | **Age (median)** | **Sex (% male)** | **Primary endpoint** | **PROM** | **Timing of evaluation** |
| Moon et al. [34] | 2021 | Monocenter, retrospective | Cohort | 1-7-2015 until 31-10-2019 | STS | 95 | 64 | 58.9 | PROMIS-scores between patients who had undergone preoperative and postoperative RT | PROMIS anxiety, depression, pain interference, physical function | Preoperative, immediate postoperative and after completion of all treatment (> 45 days) |
| Oh et al. [35] | 2018 | Monocenter, retrospective | Cohort | 2008-2014 | STS | 150 | 47 | 54 | Predictive factors that affect postoperative functional outcomes and longitudinal changes in functional outcome | TESS | Every 3 months for 1 year postoperatively, every 6 months from 2-5 years after surgery |
| **Authors** | **Year** | **Study type** | **Study design** | **Inclusion period** | **Population** | **Patients (STS cohort)** | **Age (median)** | **Sex (% male)** | **Primary endpoint** | **PROM** | **Timing of evaluation** |
| Ostacoli et al. [36] | 2014 | Multicenter, prospective | Cohort | 6-2008 until 2-2010 | STS patients undergoing chemotherapy | 56 | 53.4 | 50 | Anxiety and depression in early stages of treatment in STS patients undergoing chemotherapy and patients with common types of cancer undergoing chemotherapy | FACT-G, HADS | Within 3 months since starting chemotherapy |
| Payne et al. [37] | 2013 | Monocenter, prospective | Cohort | 1989-2008 | UE STS that required reconstruction | 88 | NR | 57.5 | Upper limb function preoperatively and 1 year postoperatively | TESS | Preoperative and 1 year postoperatively |
| Podleska et al. [38] | 2017 | Monocenter, retrospective | Cohort | 2014-2015 | ILP patients | 27 | 51.9 | 52.7 | Quality of life | EORTC-QLQ-C30, SMFA-D | Short-term follow-up (<36 months) and long-term follow-up (>36 months) |
| **Authors** | **Year** | **Study type** | **Study design** | **Inclusion period** | **Population** | **Patients (STS cohort)** | **Age (median)** | **Sex (% male)** | **Primary endpoint** | **PROM** | **Timing of evaluation** |
| Pradhan et al. [39] | 2006 | Multicenter, retrospective | Cohort | 1990-2001 | STS of the adductor compartment | 184 | 56 | 51.1 | Oncological and functional outcome | TESS | NR |
| Reichardt et al. [40] | 2012 | Multicenter | Cross-sectional | 12-2009 until 3-2011 | Metastatic STS | 99 | NR | NR | Utility weights in metastatic sarcoma patients with complete response, partial response or stable disease | EORTC-QLQ-C30, EQ-5D-3L, the 3-item Cancer-Related Symptoms Questionnaire | NA |
| Reijers et al. [41] | 2021 | Multicenter, retrospective | Cohort | 2008-2016 | Locally advanced STS | 97 | NR | 66 | Long-term quality of life after ILP + resection, extended resection, amputation and secondary amputation after ILP + resection | EORTC-QLQ-C30, CWS, HADS, TESS | 2-10 years after diagnosis |
| Saebye et al. [42] | 2020 | Monocenter, prospective | Cohort | 2015-2017 | STS | 29 | 63 | 52 | Changes in objectively measured functional outcome within the first year after surgery | TESS, WHO-5 wellbeing, HADS, ISI, MFI-20 | Prior to surgery, 1 month, 2 months, 3 months and 1 year after surgery |
| **Authors** | **Year** | **Study type** | **Study design** | **Inclusion period** | **Population** | **Patients (STS cohort)** | **Age (median)** | **Sex (% male)** | **Primary endpoint** | **PROM** | **Timing of evaluation** |
| Saebye et al. [43] | 2017 | Multicenter | Cross-sectional | 1-1-2009 until 31-12-2011 | STS treated with first-time limb-sparing surgery without bone resection | 128 | 61 | 58 | Tumor- and patient-related factors associated with functional outcome and quality of life | TESS, EORTC-QLQ-C30 | NA |
| Schreiber et al. [44] | 2006 | Monocenter | Cross-sectional | 2001-2003 | ESTS | 100 | 55 | 56 | To evaluate how functional disability impacts on the HRQoL | TESS, RNL, EQ-5D-5L (EQ-5D-5L-VAS) | At 1 year post surgery |
| Singer et al. [45] | 2023 | Multicenter | Cross-sectional | 9-2017 until 2-2020 | STS | 340 | 58.6 | 56.2 | Health-related quality of life in patients undergoing adjuvant versus neo-adjuvant radiotherapy | EORTC-QLQ-c30 | After radiotherapy |
| Slump et al. [46] | 2018 | Monocenter, prospective | Cohort | 1-2006 until 1-2015 | ESTS | 266 | 59.2 | 55 | Complication rates and functional outcomes of free and pedicled flaps | TESS | Preoperative and postoperative (9-12 months) |
| Tanaka et al. [47] | 2016 | Monocenter, retrospective | Cohort | 2002-2014 | STS in the anterior compartment of the thigh | 18 | 62 | 77.8 | Knee extension strength after surgery for STS in the anterior compartment of the thigh | TESS, EQ-5D-3L, SF-8 | Median follow-up 46.0 months |
| **Authors** | **Year** | **Study type** | **Study design** | **Inclusion period** | **Population** | **Patients (STS cohort)** | **Age (median)** | **Sex (% male)** | **Primary endpoint** | **PROM** | **Timing of evaluation** |
| Tanaka et al. [48] | 2017 | Monocenter, retrospective | Cohort | 1991-2005 | LE STS | 17 | 54 | 47 | The decrease in muscle strength as a result of muscle resection | TESS, EQ-5D-3L, SF-8 | NR |
| Tanaka et al. [49] | 2023 | Monocenter, prospective | Cohort | 2014-2019 | STS of the thigh | 15 | 47 | 53.3 | Muscle strength and functional outcomes | TESS, EQ-5D | Preoperatively and at 3, 6, 12, 18 and 24 months postoperatively |
| Thijssens et al. [50] | 2006 | Monocenter, prospective | Cohort | 1991-2003 | Locally advanced STS | 39 | 59 | 41 | Quality of life after treatment for locally advanced STS | SF-36, IES | Long-term follow-up (median time since perfusion 7 years) |
| Townley et al. [51] | 2013 | Monocenter, prospective | Cohort | 2007-2012 | Extremity or truncal STS patients requiring a free flap | 32 | 57 | NR | The experience with free flap microsurgical reconstruction of irradiated STS defects | TESS | After completing at least 1 year follow-up |
| Turcotte et al. [52] | 2009 | Monocenter, prospective | Cohort | 1994-2005 | Popliteal sarcomas | 18 | 54 | 33.3 | Oncological and functional outcome | TESS | NR |
| **Authors** | **Year** | **Study type** | **Study design** | **Inclusion period** | **Population** | **Patients (STS cohort)** | **Age (median)** | **Sex (% male)** | **Primary endpoint** | **PROM** | **Timing of evaluation** |
| Werenski et al. [53] | 2024 | Monocenter, prospective | Cohort | 2016-2022 | Myxofibrosarcoma with surgical management as either primary tumor resection or tumor bed excision after surgery at an outside hospital | 79 | 69 | 63 | PROs of physical function, mental function and visual analogue scale for pain after sarcoma resection | PROMIS Global Health Short Form-Mental, PROMIS Global Health Short Form-Physical, PROMIS Physical Function Short-Form 10a | Preoperatively, at 1, 2, 6 months, 1 and 2 years postoperatively |
| Wilke et al. [54] | 2019 | Monocenter, prospective | Cohort | 1-9-2016 until 31-12-2016 | Nonmetastatic sarcoma | 83 | 63 | 51 | PROMIS scores of patients who had an unplanned resection versus planned resection | PROMIS physical function, anxiety, depression, fatigue, sleep disturbance, ability to participate, pain interference | Postoperative (< 1 year following surgery and > 1 year following surgery) |
| **Authors** | **Year** | **Study type** | **Study design** | **Inclusion period** | **Population** | **Patients (STS cohort)** | **Age (median)** | **Sex (% male)** | **Primary endpoint** | **PROM** | **Timing of evaluation** |
| Willis et al. [55] | 2023 | Monocenter | Cross-sectional | 10-2021 until 12-2021 | Patients who underwent resection of primary, recurrent or metastasized RPS | 127 | 62 | 51.2 | To analyze patient-reported outcomes after resection of RPS | EORTC-QLQ-c30, WEMWBS, FoP-Q-SF, Pro-CTCAE | Median interval between first diagnosis and completion of questionnaires 80 months (IQR 33-140), between last operation and completion of questionnaires 31 months (IQR 12-73 months) |
| Wong et al. [56] | 2017 | NR, prospective | Cohort | 1998-2012 | RPS patients that had undergone preoperative RT and surgery | 48 | NR | NR | The effect of treatment-related toxicities on quality of life | EORTC-QLQ-C30 | Pretreatment, 1 month post-RT, 6, 12 months or any point following RT (during follow-up) |
| Wright et al. [57] | 2008 | Monocenter, retrospective | Cohort | 1997-2005 | UE sarcomas | 43 | NR | NR | Oncological and functional outcome | TESS | After discharge from hospital |
| **Authors** | **Year** | **Study type** | **Study design** | **Inclusion period** | **Population** | **Patients (STS cohort)** | **Age (median)** | **Sex (% male)** | **Primary endpoint** | **PROM** | **Timing of evaluation** |
| Younger et al. [58] | 2021 | Multicenter, prospective | Cohort | NR | Advanced STS | 137 | 62 | 49.6 | Priorities towards quality versus length of life | EORTC QLQ-C30 | Baseline to post-cycle 4 of chemotherapy; in the current study only baseline |
| Zhuang et al. [59] | 2022 | Monocenter, retrospective | Cohort | 8-2009 until 12-2021 | RPS patients that underwent curative surgery | 161 | 55.2 | 47.2 | Changes in quality of life of patients with multivisceral resection compared to simple tumor resection after surgery | EORTC QLQ-C30 | 0-6 months; 6-12 months; 12-18 months; 18-36 months; 2-5 years; > 5 years |

*Abbreviations. STS: soft-tissue sarcoma. PROM: patient-reported outcome measure. UE: upper extremity. RPS: retroperitoneal sarcoma. NR: not reported. ESTS: extremity soft-tissue sarcoma. NA: not applicable. LE: lower extremity. RT: radiotherapy. HRQoL: health-related quality of life. ILP: isolated limb perfusion. PRO: patient-reported outcome.*

**References**

1. Ahmed, S. K., Kaggal, S., Harmsen, W. S., Sawyer, J. W., Houdek, M. T., Rose, P. S., & Petersen, I. A. (2021). Patient-reported functional outcomes in a cohort of hand and foot sarcoma survivors treated with limb sparing surgery and radiation therapy. *Journal of Surgical Oncology*, *123*(1), 110–116. https://doi.org/10.1002/jso.26258

2. Andersen, N. J., Mate, K. K. B., Bergeron, C., Turcotte, R., & Körner, A. (2024). Evaluating health perceptions of soft-tissue sarcoma patients using the Wilson-Cleary Model to identify key targets for improving outcomes and quality of care. *Surgical Oncology*, *52*(November 2023), 1–7. https://doi.org/10.1016/j.suronc.2023.102028

3. Baysal, Ö., Toprak, C. Ş., Günar, B., & Erol, B. (2021). Soft tissue sarcoma of the upper extremity: oncological and functional results after surgery. *Journal of Hand Surgery: European Volume*, *46*(6), 659–664. https://doi.org/10.1177/1753193421998252

4. Brandes, F., Striefler, J. K., Dörr, A., Schmiester, M., Märdian, S., Koulaxouzidis, G., … Flörcken, A. (2021). Impact of a specialised palliative care intervention in patients with advanced soft tissue sarcoma – a single-centre retrospective analysis. *BMC Palliative Care*, *20*(1), 1–9. https://doi.org/10.1186/s12904-020-00702-1

5. Callegaro, D., Miceli, R., Brunelli, C., Colombo, C., Sanfilippo, R., Radaelli, S., … Fiore, M. (2015). Long-term morbidity after multivisceral resection for retroperitoneal sarcoma. *British Journal of Surgery*, *102*(9), 1079–1087. https://doi.org/10.1002/bjs.9829

6. Cassidy, R. J., Indelicato, D. J., Gibbs, C. P., Scarborough, M. T., Morris, C. G., & Zlotecki, R. A. (2016). Function Preservation After Conservative Resection and Radiotherapy for Soft-tissue Sarcoma of the Distal Extremity: Utility and Application of the Toronto Extremity Salvage Score (TESS). *American Journal of Clinical Oncology: Cancer Clinical Trials*, *39*(6), 600–603. https://doi.org/10.1097/COC.0000000000000107

7. Cribb, G. L., Loo, S. C. S., & Dickinson, I. (2010). Limb salvage for soft-tissue sarcomas of the foot and ankle. *Journal of Bone and Joint Surgery - Series B*, *92*(3), 424–429. https://doi.org/10.1302/0301-620X.92B3.22331

8. Penel, N., Coindre, J., Giraud, A., & Terrier, P. (2018). Presentation and Outcome of Frequent and Rare Sarcoma Histologic Subtypes : A Study of 10 , 262 Patients With Localized Visceral / Soft Tissue Sarcoma Managed in Reference Centers, 1179–1187. https://doi.org/10.1002/cncr.31176

9. Davidge, K. M., Wunder, J., Tomlinson, G., Wong, R., Lipa, J., & Davis, A. M. (2010). Function and health status outcomes following soft tissue reconstruction for limb preservation in extremity soft tissue sarcoma. *Annals of Surgical Oncology*, *17*(4), 1052–1062. https://doi.org/10.1245/s10434-010-0915-5

10. Davidson, D., Barr, R. D., Riad, S., Griffin, A. M., Chung, P. W., Catton, C. N., … Wunder, J. S. (2016). Health-related quality of life following treatment for extremity soft tissue sarcoma. *Journal of Surgical Oncology*, *114*(7), 821–827. https://doi.org/10.1002/jso.24424

11. Davis, A. M., Sennik, S., Griffin, A. M., Wunder, J. S., O’Sullivan, B., Catton, C. N., & Bell, R. S. (2000). Predictors of functional outcomes following limb salvage surgery for lower-extremity soft tissue sarcoma. *Journal of Surgical Oncology*, *73*(4), 206–211. https://doi.org/10.1002/(SICI)1096-9098(200004)73:4<206::AID-JSO4>3.0.CO;2-5

12. Drabbe, C., Van der Graaf, W. T. A., De Rooij, B. H., Grünhagen, D. J., Soomers, V. L. M. N., Van de Sande, M. A. J., … Husson, O. (2021). The age-related impact of surviving sarcoma on health-related quality of life: data from the SURVSARC study. *ESMO Open*, *6*(1), 100047. https://doi.org/10.1016/j.esmoop.2021.100047

13. Eichler, M., Hentschel, L., Richter, S., Hohenberger, P., Kasper, B., Andreou, D., … Schuler, M. K. (2020). The health-related quality of life of sarcoma patients and survivors in germany—cross-sectional results of a nationwide observational study (Prosa). *Cancers*, *12*(12), 1–19. https://doi.org/10.3390/cancers12123590

14. Fiore, M., Brunelli, C., Miceli, R., Manara, M., Lenna, S., Rampello, N. N., … Gronchi, A. (2020). A Prospective Observational Study of Multivisceral Resection for Retroperitoneal Sarcoma: Clinical and Patient-Reported Outcomes 1 Year After Surgery. *Annals of Surgical Oncology*, 3904–3916. https://doi.org/10.1245/s10434-020-09307-7

15. Fischer, S., Soimaru, S., Hirsch, T., Kueckelhaus, M., Seitz, C., Lehnhardt, M., … Daigeler, A. (2015). Local tendon transfer for knee extensor mechanism reconstruction after soft tissue sarcoma resection. *Journal of Plastic, Reconstructive and Aesthetic Surgery*, *68*(5), 729–735. https://doi.org/10.1016/j.bjps.2015.01.002

16. Gerrand, C. H., Wunder, J. S., Kandel, R. A., O’Sullivan, B., Catton, C. N., Bell, R. S., … Davis, A. M. (2004). The influence of anatomic location on functional outcome in lower-extremity soft-tissue sarcoma. *Annals of Surgical Oncology*, *11*(5), 476–482. https://doi.org/10.1245/ASO.2004.07.016

17. Ghert, M. A., Davis, A. M., Griffin, A. M., Alyami, A. H., White, L., Kandel, R. A., … Wunder, J. S. (2005). The surgical and functional outcome of limb-salvage surgery with vascular reconstruction for soft tissue sarcoma of the extremity. *Annals of Surgical Oncology*, *12*(12), 1102–1110. https://doi.org/10.1245/ASO.2005.06.036

18. Götzl, R., Sterzinger, S., Semrau, S., Vassos, N., Hohenberger, W., Grützmann, R., … Beier, J. P. (2019). Patient’s quality of life after surgery and radiotherapy for extremity soft tissue sarcoma-a retrospective single-center study over ten years. *Health and Quality of Life Outcomes*, *17*(1), 1–10. https://doi.org/10.1186/s12955-019-1236-4

19. Gough, N., Koffman, J., Ross, J. R., Riley, J., & Judson, I. (2017). Symptom Burden in Advanced Soft-Tissue Sarcoma. *Journal of Pain and Symptom Management*, *53*(3), 588–597. https://doi.org/10.1016/j.jpainsymman.2016.10.357

20. Gough, N., Koffman, J., Ross, J. R., Riley, J., & Judson, I. (2019). Does palliative chemotherapy really palliate and are we measuring it correctly? A mixed methods longitudinal study of health related quality of life in advanced soft tissue sarcoma. *PLoS ONE*, *14*(9), 1–24. https://doi.org/10.1371/journal.pone.0210731

21. Heaver, C., Isaacson, A., Gregory, J. J., Cribb, G., & Cool, P. (2016). Patient factors affecting the Toronto extremity salvage score following limb salvage surgery for bone and soft tissue tumors. *Journal of Surgical Oncology*, *113*(7), 804–810. https://doi.org/10.1002/jso.24247

22. Homsy, P., Kantonen, I., Salo, J., Albäck, A., & Tukiainen, E. (2022). Reconstruction of the superficial femoral vessels with muscle flap coverage for soft tissue sarcomas of the proximal thigh. *Microsurgery*, *42*(6), 568–576. https://doi.org/10.1002/micr.30932

23. Hoven-Gondrie, M. L., Thijssens, K. M. J., Geertzen, J. H. B., Pras, E., Van Ginkel, R. J., & Hoekstra, H. J. (2008). Isolated limb perfusion and external beam radiotherapy for soft tissue sarcomas of the extremity: Long-term effects on normal tissue according to the LENT-SOMA scoring system. *Annals of Surgical Oncology*, *15*(5), 1502–1510. https://doi.org/10.1245/s10434-008-9850-0

24. Jakob, J., Hentschel, L., Richter, S., Kreisel, I., Hohenberger, P., Kasper, B., … Eichler, M. (2022). Transferability of Health-Related Quality of Life Data of Large Observational Studies to Clinical Practice: Comparing Retroperitoneal Sarcoma Patients from the PROSa Study to a TARPS-WG Cohort. *Oncology Research and Treatment*, *45*(11), 660–669. https://doi.org/10.1159/000525288

25. Jones, K. B., Ferguson, P. C., Deheshi, B., Riad, S., Griffin, A., Bell, R. S., & Wunder, J. S. (2010). Complete Femoral Nerve Resection with Soft Tissue Sarcoma : Functional Outcomes, 401–406. https://doi.org/10.1245/s10434-009-0745-5

26. Kapoor, T., Banuelos, J., Adabi, K., Moran, S. L., & Manrique, O. J. (2018). Analysis of clinical outcomes of upper and lower extremity reconstructions in patients with soft-tissue sarcoma. *Journal of Surgical Oncology*, *118*(4), 614–620. https://doi.org/10.1002/jso.25201

27. Kasper, B., Pink, D., Rothermundt, C., Richter, S., Augustin, M., Kollar, A., … Schuler, M. K. (2024). Geriatric Assessment of Older Patients Receiving Trabectedin in First-Line Treatment for Advanced Soft Tissue Sarcomas: The E-TRAB Study from The German Interdisciplinary Sarcoma Group (GISG-13). *Cancers*, *16*(3). https://doi.org/10.3390/cancers16030558

28. Kemp, M. A., Hinsley, D. E., Gwilym, S. E., Giele, H. P., Athanasou, N. A., & Gibbons, C. L. (2011). Functional and oncological outcome following marginal excision of well-differentiated forearm Liposarcoma with nerve involvement. *Journal of Hand Surgery*, *36*(1), 94–100. https://doi.org/10.1016/j.jhsa.2010.09.032

29. Kokkali, S., Boukovinas, I., Samantas, E., Papakotoulas, P., Athanasiadis, I., Andreadis, C., … Psyrri, A. (2022). A Multicenter, Prospective, Observational Study to Assess the Clinical Activity and Impact on Symptom Burden and Patients’ Quality of Life in Patients with Advanced Soft Tissue Sarcomas Treated with Trabectedin in a Real-World Setting in Greece. *Cancers*, *14*(8). https://doi.org/10.3390/cancers14081879

30. Komatsu, S., Okamoto, M., Shiba, S., Kaminuma, T., Okazaki, S., Kiyohara, H., … Ohno, T. (2021). Prospective evaluation of quality of life and functional outcomes after carbon ion radiotherapy for inoperable bone and soft tissue sarcomas. *Cancers*, *13*(11). https://doi.org/10.3390/cancers13112591

31. Kruiswijk, A. A., van de Sande, M. A. J., Verhoef, C., Schrage, Y. M., Haas, R. L., Bemelmans, M. H. A., … van Bodegom-Vos, L. (2024). Changes in Health-Related Quality of Life following Surgery in Patients with High-Grade Extremity Soft-Tissue Sarcoma: A Prospective Longitudinal Study. *Cancers*, *16*(3). https://doi.org/10.3390/cancers16030547

32. Lim, H. J., Ong, C. A. J., Skanthakumar, T., Mak, L. Y. H., Wasudevan, S. D., Tan, J. W. S., … Teo, M. C. C. (2020). Retrospective quality of life study in patients with retroperitoneal sarcoma in an Asian population. *Health and Quality of Life Outcomes*, *18*(1), 1–9. https://doi.org/10.1186/s12955-020-01491-0

33. Macarthur, I. R., McInnes, C. W., Dalke, K. R., Akra, M., Banerji, S., Buchel, E. W., & Hayakawa, T. J. (2019). Patient Reported Outcomes Following Lower Extremity Soft Tissue Sarcoma Resection with Microsurgical Preservation of Ambulation. *Journal of Reconstructive Microsurgery*, *35*(3), 168–175. https://doi.org/10.1055/s-0038-1668116

34. Moon, T. M., Furdock, R., Rhea, L., Pergolotti, M., Cipriano, C., & Spraker, M. B. (2021). PROMIS scores of patients undergoing neoadjuvant and adjuvant radiation therapy for surgically excised soft tissue sarcoma. *Clinical and Translational Radiation Oncology*, *31*(August), 42–49. https://doi.org/10.1016/j.ctro.2021.08.008

35. Oh, E., Seo, S. W., & Han, K. J. (2018). A longitudinal study of functional outcomes in patients with limb salvage surgery for soft tissue sarcoma. *Sarcoma*, *2018*. https://doi.org/10.1155/2018/6846275

36. Ostacoli, L., Saini, A., Zuffranieri, M., Boglione, A., Carletto, S., De Marco, I., … Comandone, A. (2014). Quality of Life, Anxiety and Depression in Soft Tissue Sarcomas as Compared to More Common Tumours: An Observational Study. *Applied Research in Quality of Life*, *9*(1), 123–131. https://doi.org/10.1007/s11482-013-9213-2

37. Payne, C. E., Hofer, S. O. P., Zhong, T., Griffin, A. C., Ferguson, P. C., & Wunder, J. S. (2013). Functional outcome following upper limb soft tissue sarcoma resection with flap reconstruction. *Journal of Plastic, Reconstructive and Aesthetic Surgery*, *66*(5), 601–607. https://doi.org/10.1016/j.bjps.2013.01.034

38. Podleska, L. E., Kaya, N., Farzaliyev, F., Pöttgen, C., Bauer, S., & Taeger, G. (2017). Lower limb function and quality of life after ILP for soft-tissue sarcoma. *World Journal of Surgical Oncology*, *15*(1), 1–10. https://doi.org/10.1186/s12957-017-1150-3

39. Pradhan, A., Cheung, Y. C., Grimer, R. J., Abudu, A., Peake, D., Ferguson, P. C., … Sim, F. H. (2006). Does the method of treatment affect the outcome in soft-tissue sarcomas of the adductor compartment? *Journal of Bone and Joint Surgery - Series B*, *88*(11), 1480–1486. https://doi.org/10.1302/0301-620X.88B11.17424

40. Reichardt, P., Leahy, M., Garcia Del Muro, X., Ferrari, S., Martin, J., Gelderblom, H., … Blay, J. Y. (2012). Quality of life and utility in patients with metastatic soft tissue and bone sarcoma: The sarcoma treatment and burden of illness in North America and Europe (SABINE) study. *Sarcoma*, *2012*. https://doi.org/10.1155/2012/740279

41. Reijers, S. J. M., Husson, O., Soomers, V. L. M. N., Been, L. B., Bonenkamp, J. J., van de Sande, M. A. J., … van Houdt, W. J. (2022). Health-related quality of life after isolated limb perfusion compared to extended resection, or amputation for locally advanced extremity sarcoma: Is a limb salvage strategy worth the effort? *European Journal of Surgical Oncology*, *48*(3), 500–507. https://doi.org/10.1016/j.ejso.2021.08.007

42. Saebye, C., Amidi, A., Keller, J., Andersen, H., & Baad-Hansen, T. (2020). Changes in functional outcome and quality of life in soft tissue sarcoma patients within the first year after surgery: A prospective observational study. *Cancers*, *12*(2). https://doi.org/10.3390/cancers12020463

43. Saebye, C., Fugloe, H. M., Nymark, T., Safwat, A., Petersen, M. M., Baad-Hansen, T., … Keller, J. (2017). Factors associated with reduced functional outcome and quality of life in patients having limb-sparing surgery for soft tissue sarcomas–a national multicenter study of 128 patients. *Acta Oncologica*, *56*(2), 239–244. https://doi.org/10.1080/0284186X.2016.1268267

44. Schreiber, D., Bell, R. S., Wunder, J. S., O’Sullivan, B., Turcotte, R., Masri, B. A., & Davis, A. M. (2006). Evaluating function and health related quality of life in patients treated for extremity soft tissue sarcoma. *Quality of Life Research*, *15*(9), 1439–1446. https://doi.org/10.1007/s11136-006-0001-4

45. Singer, S., Semrau, S., Golcher, H., Fechner, K., Kallies, A., Zapata Bonilla, S., … Eichler, M. (2023). The health-related quality of life of sarcoma patients treated with neoadjuvant versus adjuvant radiotherapy – Results of a multi-center observational study. *Radiotherapy and Oncology*, *189*(September). https://doi.org/10.1016/j.radonc.2023.109913

46. Slump, J., Hofer, S. O. P., Ferguson, P. C., Wunder, J. S., Griffin, A. M., Hoekstra, H. J., … O’Neill, A. C. (2018). Flap choice does not affect complication rates or functional outcomes following extremity soft tissue sarcoma reconstruction. *Journal of Plastic, Reconstructive and Aesthetic Surgery*, *71*(7), 989–996. https://doi.org/10.1016/j.bjps.2018.04.002

47. Tanaka, A., Yoshimura, Y., Aoki, K., Kito, M., Okamoto, M., Suzuki, S., … Kato, H. (2016). Knee extension strength and post-operative functional prediction in quadriceps resection for soft-tissue sarcoma of the thigh. *Bone and Joint Research*, *5*(6), 232–238. https://doi.org/10.1302/2046-3758.56.2000631

48. Tanaka, A., Yoshimura, Y., Aoki, K., Okamoto, M., Kito, M., Suzuki, S., … Kato, H. (2017). Prediction of muscle strength and postoperative function after knee flexor muscle resection for soft tissue sarcoma of the lower limbs. *Orthopaedics and Traumatology: Surgery and Research*, *103*(7), 1081–1085. https://doi.org/10.1016/j.otsr.2017.07.005

49. Tanaka, A., Okamoto, M., Kito, M., Yoshimura, Y., Aoki, K., Suzuki, S., … Takahashi, J. (2023). Muscle strength and functional recovery for soft-tissue sarcoma of the thigh: a prospective study. *International Journal of Clinical Oncology*, *28*(7), 922–927. https://doi.org/10.1007/s10147-023-02348-4

50. Thijssens, K. M. J., Hoekstra-Weebers, J. E. H. M., Van Ginkel, R. J., & Hoekstra, H. J. (2006). Quality of life after hyperthermic isolated limb perfusion for locally advanced extremity soft tissue sarcoma. *Annals of Surgical Oncology*, *13*(6), 864–871. https://doi.org/10.1245/ASO.2006.05.023

51. Townley, W. A., Mah, E., Neill, A. C. O., Wunder, J. S., Ferguson, P. C., Zhong, T., & Hofer, S. O. P. (2013). Reconstruction of sarcoma defects following pre-operative radiation : Free tissue transfer is safe and reliable. *British Journal of Plastic Surgery*, *66*(11), 1575–1579. https://doi.org/10.1016/j.bjps.2013.06.029

52. Turcotte, R. E., Ferrone, M., Lsler, M. H., & Wong, C. (2009). Outcomes in patients with popliteal sarcomas. *Canadian Journal of Surgery*, *52*(1), 51–55. https://doi.org/10.1016/s0276-1092(09)79545-8

53. Werenski, J. O., Gonzalez, M. R., Fourman, M. S., Hung, Y. P., & Lozano-Calderón, S. A. (2024). Does Wound VAC Temporization Offer Patient-Reported Outcomes Similar to Single-Stage Excision Reconstruction After Myxofibrosarcoma Resection? *Annals of Surgical Oncology*, *31*(4), 2757–2765. https://doi.org/10.1245/s10434-023-14839-9

54. Wilke, B. K., Cooper, A. R., Aratani, A. K., Scarborough, M. T., Gibbs, C. P., & Spiguel, A. (2019). Evaluation of planned versus unplanned soft-tissue sarcoma resection using PROMIS measures. *Sarcoma*, *2019*. https://doi.org/10.1155/2019/1342615

55. Willis, F., Buck, L., Musa, J., Hinz, U., Mechtersheimer, G., Seidensaal, K., … Schneider, M. (2023). Long-term quality of life after resection of retroperitoneal soft tissue sarcoma. *European Journal of Surgical Oncology*, *49*(11). https://doi.org/10.1016/j.ejso.2023.07.003

56. Wong, P., Kassam, Z., Springer, A. N., Gladdy, R., Chung, P., Ringash, J., & Catton, C. (2017). Long-Term Quality of Life of Retroperitoneal Sarcoma Patients Treated with Pre-Operative Radiotherapy and Surgery. *Cureus*, *9*(10). https://doi.org/10.7759/cureus.1764

57. Wright, E. H. C., Gwilym, S., Gibbons, C. L. M. H., Critchley, P., & Giele, H. P. (2008). Functional and oncological outcomes after limb-salvage surgery for primary sarcomas of the upper limb. *Journal of Plastic, Reconstructive and Aesthetic Surgery*, *61*(4), 382–387. https://doi.org/10.1016/j.bjps.2007.01.080

58. Younger, E., Husson, O., Bennister, L., Whelan, J., Wilson, R., Roast, A., … van der Graaf, W. T. (2018). Age-related sarcoma patient experience: results from a national survey in England. *BMC Cancer*, *18*(1), 1–11. https://doi.org/10.1186/s12885-018-4866-8

59. Zhuang, A., Fang, Y., Ma, L., Yang, H., Lu, W., Zhou, Y., … Tong, H. (2022). Does Aggressive Surgery Mean Worse Quality of Life and Functional Capacity in Retroperitoneal Sarcoma Patients?—A Retrospective Study of 161 Patients from China. *Cancers*, *14*(20), 1–13. https://doi.org/10.3390/cancers14205126
