# Supplementary material for: Twenty-five years of experience with patient-reported outcome measures in soft-tissue sarcoma patients: a systematic review
Source: Qual Life Res. 2024 Sep 11;33(12):3189–211. doi: 10.1007/s11136-024-03755-4 (PMC11599342; doi:10.1007/s11136-024-03755-4)
Supplement: Supplementary file 6 — Supplementary file6 (DOCX 16 KB) [file 11136_2024_3755_MOESM6_ESM.docx]

**Supplementary Information 6.** Identified patient-reported outcome measures

| **Patient-reported outcome measures (n = 39)** | **Frequency of PROM^a^ used in the included studies, n = 59 (%)** |
| --- | --- |
| Toronto Extremity Salvage Score (TESS) | 28 (47.5) |
| European Organization for Research and Treatment of Cancer Quality of Life Questionnaire-Core 30-questions (EORTC-QLQ-C30) | 17 (28.8) |
| Euro – Quality of Life – 5 Dimensions – 3 Levels (EQ-5D-3L) | 5 (8.5) |
| Short Form Health Survey (SF-36) | 4 (6.8) |
| Patient-Reported Outcome Measurement Information System – Physical Function (PROMIS – Physical Function) | 4 (6.8) |
| Euro – Quality of Life – 5 Dimensions – 5 Levels (EQ-5D-5L) | 4 (6.8) |
| Lower Extremity Functional Scale (LEFS) | 3 (5.1) |
| Short Form 8 (SF-8) | 3 (5.1) |
| Hospital Anxiety and Depression Scale (HADS) | 3 (5.1) |
| Patient-Reported Outcome Measurement Information System – Depression (PROMIS – Depression) | 3 (5.1) |
| Patient-Reported Outcome Measurement Information System – Pain Interference (PROMIS – Pain Interference) | 3 (5.1) |
| Re-integration to Normal Living (RNL) | 3 (5.1) |
| Disabilities of the Arm, Shoulder and Hand (QuickDASH) | 2 (3.4) |
| Brief Pain Inventory – Short Form (BPI-SF) | 2 (3.4) |
| Patient-Reported Outcome Measurement Information System – Anxiety (PROMIS – Anxiety) | 2 (3.4) |
| Patient-Reported Outcomes version of the Common Terminology Criteria for Adverse Events (PRO-CTCAE) | 2 (3.4) |
| Michigan Hand Outcomes Survey (MHQ) | 1 (1.7) |
| Foot and Ankle Outcomes Survey (FAOS) | 1 (1.7) |
| Patient-Reported Outcome Measurement Information System – Fatigue (PROMIS – Fatigue) | 1 (1.7) |
| Patient-Reported Outcome Measurement Information System – Sleep disturbance (PROMIS – Sleep disturbance) | 1 (1.7) |
| Patient-Reported Outcome Measurement Information System – Ability to participate (PROMIS – Ability to participate) | 1 (1.7) |
| Late Effects Normal Tissues-Subjective, Objective, Management, Analytic (LENT-SOMA) | 1 (1.7) |
| Short Musculoskeletal Function Assessment (SMFA) | 1 (1.7) |
| Cancer Worry Scale (CWS) | 1 (1.7) |
| World Health Organization Five Wellbeing Index (WHO-5) | 1 (1.7) |
| Insomnia Severity Index (ISI) | 1 (1.7) |
| Multidimensional Fatigue Inventory (MFI-20) | 1 (1.7) |
| Impact of Event Scale (IES) | 1 (1.7) |
| Numeric Rating Scale (NRS) | 1 (1.7) |
| National Comprehensive Cancer Network (NCCN) Distress Thermometer | 1 (1.7) |
| Minimal Documentation System (MIDOS) | 1 (1.7) |
| M.D. Anderson Symptom Inventory (MDASI) | 1 (1.7) |
| Memorial Symptom Assessment Scale-Short Form (MSAS-SF) | 1 (1.7) |
| Three-item Cancer-Related Symptoms Questionnaire | 1 (1.7) |
| Functional Assessment of Cancer Therapy (FACT-G) | 1 (1.7) |
| Functional Assessment of Chronic Illness Therapy - Fatigue (FACIT-F) | 1 (1.7) |
| Patient-Reported Outcome Measurement Information System - Global Health (PROMIS - Global Health) | 1 (1.7) |
| The Warwick-Edinburgh Mental Wellbeing Scales (WEMWBS) | 1 (1.7) |
| Fear of Progression Questionnaire-Short Form (FoP-Q-SF) | 1 (1.7) |

*Abbreviations. ^a^PROM: patient-reported outcome measure.*
