# Supplementary material for: Twenty-five years of experience with patient-reported outcome measures in soft-tissue sarcoma patients: a systematic review
Source: Qual Life Res. 2024 Sep 11;33(12):3189–211. doi: 10.1007/s11136-024-03755-4 (PMC11599342; doi:10.1007/s11136-024-03755-4)
Supplement: Supplementary file 7 — Supplementary file1 (DOCX 16 KB) [file 11136_2024_3755_MOESM7_ESM.docx]

**Supplementary Information**. Search strategies MEDLINE

**Search strategy 1: Identification of PROMs**

("Sarcoma"[Mesh] OR sarcoma*[tiab] OR musculoskeletal tumo*[tiab])

AND

("Patient Reported Outcome Measures"[Mesh] OR Patient Reported[tiab] OR promis[tiab] OR "Mental Health"[Mesh] OR "Anxiety"[Mesh] OR "Depression"[Mesh] OR mental health[tiab] OR "Health status"[Mesh] OR health status[tiab] OR anxiet*[tiab] OR depress*[tiab] OR social health[tiab] OR psychosocial*[tiab] OR psycho-social*[tiab] OR physical*[tiab] OR participat*[tiab] OR wellbeing*[tiab] OR "Morbidity"[Mesh] OR morbidit*[tiab] OR "Quality of Life"[Mesh] OR Quality of Life[tiab] OR life quality[tiab] OR qol[tiab] OR hrqol[tiab] OR questionnaire*[tiab]) AND ("Cohort Studies"[Mesh] OR cohort[tiab] OR follow-up[tiab] OR prospective[tiab] OR observational[tiab] OR longitudinal[tiab])

NOT

(("Child"[Mesh] OR "Adolescent"[Mesh] OR "Infant"[Mesh]) NOT "Adult"[Mesh]) NOT (bone sarcoma[ti] OR "Osteosarcoma"[Mesh])

**Search strategy 2: Methodological evaluation of PROMs**

("Sarcoma"[Mesh] OR sarcoma*[tiab] OR musculoskeletal tumo*[tiab])

AND

("Toronto extremity salvage score*" OR tess*[tiab] OR "EORTC-QLQ-C30" OR "EORTC quality of life questionnaire" OR "EQ-5D-3L" OR "Short Form Health Survey*" OR "SF-36" OR "Lower Extremity Functional Scale*" OR LEFS[tiab] OR "Short Form 8" OR "SF-8" OR "Hospital Anxiety and Depression Scale*" OR HADS[tiab] OR "Patient-Reported Outcome Measurement Information System" OR "Disabilities of the Arm, Shoulder and Hand*" OR QuickDASH*[tiab] OR "Brief Pain Inventory Short Form*" OR "BPI-SF" OR RNL[tiab] OR rinl[tiab] OR "EuroQol five-dimension" OR "EQ-5D-5L" OR "Michigan Hand Outcomes" OR MHQ[tiab] OR "Foot and Ankle Outcomes" OR FAOS[tiab] OR PROMIS[tiab] OR "Patient-Reported Outcome Measurement System*" OR Late Effects Normal Tissues-Subjective, Objective, Management[tiab] OR "LENT-SOMA" OR "Short Musculoskeletal Function Assessment*" OR SMFA[tiab] OR "Cancer Worry Scale*" OR CWS[tiab] OR "World Health Organization Five Well being Index*" OR "WHO-5" OR "Insomnia Severity Index*" OR ISI[tiab] OR Multidimensional Fatigue Inventory[tiab] OR MFI20[tiab] OR Impact Event Scale[tiab] OR NCCN Distress Thermometer[tiab] OR National Comprehensive Cancer Network Distress Thermometer[tiab] OR Minimal Documentation System[tiab] OR MIDOS[tiab] OR MDASI[tiab] OR MSAS SF[tiab] OR Three item Cancer Related Symptoms Questionnaire[tiab] OR Functional Assessment of Cancer Therapy[tiab] OR FACT G[tiab] OR “MD Anderson Symptom Inventory” OR “Memorial Symptom Assessment Scale Short Form” OR "Functional Assessment of Chronic Illness Therapy – Fatigue" OR "FACIT-F" OR "facit-fatigue" OR facit-f[tiab] OR "Patient-Reported Outcomes version of*" OR "pro-ctcae" OR "proctcae" OR proctcae[tiab] OR "warwick edinburgh mental wellbeing scale" OR "warwick edinburgh mental*" OR "wemwbs" OR "Fear of Progression Questionnaire Short Form"[tiab:~0] OR "fop-q-sf")

AND

("instrumentation" [Subheading] OR "methods" [Subheading] OR "Validation Study" [Publication Type] OR “Comparative Study”[pt] OR “psychometrics”[MeSH] OR psychometr*[tiab] OR clinimetr*[tw] OR clinometr*[tw] OR "Outcome Assessment, Health Care"[Mesh] OR “outcome assessment”[tiab] OR “outcome measure*”[tw] OR “observer variation”[MeSH] OR “observer variation”[tiab] OR “Health Status Indicators”[Mesh] OR “reproducibility of results”[MeSH] OR reproducib*[tiab] OR “discriminant analysis”[MeSH] OR reliab*[tiab] OR unreliab*[tiab] OR valid*[tiab] OR “coefficient of variation”[tiab] OR coefficient[tiab] OR homogeneity[tiab] OR homogeneous[tiab] OR “internal consistency”[tiab] OR (cronbach*[tiab] AND (alpha[tiab] OR alphas[tiab])) OR (item[tiab] AND (correlation*[tiab] OR selection*[tiab] OR reduction*[tiab])) OR agreement[tw] OR precision[tw] OR imprecision[tw] OR “precise values”[tw] OR test-retest[tiab] OR (test[tiab] AND retest[tiab]) OR (reliab*[tiab] AND (test[tiab] OR retest[tiab])) OR stability[tiab] OR interrater[tiab] OR inter-rater[tiab] OR intrarater[tiab] OR intra-rater[tiab] OR intertester[tiab] OR inter-tester[tiab] OR intratester[tiab] OR intra-tester[tiab] OR interobserver[tiab] OR inter-observer[tiab] OR intraobserver[tiab] OR intra-observer[tiab] OR intertechnician[tiab] OR inter-technician[tiab] OR intratechnician[tiab] OR intra-technician[tiab] OR interexaminer[tiab] OR inter-examiner[tiab] OR intraexaminer[tiab] OR intra-examiner[tiab] OR interassay[tiab] OR inter-assay[tiab] OR intraassay[tiab] OR intra-assay[tiab] OR interindividual[tiab] OR inter-individual[tiab] OR intraindividual[tiab] OR intra-individual[tiab] OR interparticipant[tiab] OR inter-participant[tiab] OR intraparticipant[tiab] OR intra-participant[tiab] OR kappa[tiab] OR kappa’s[tiab] OR kappas[tiab] OR repeatab*[tw] OR ((replicab*[tw] OR repeated[tw]) AND (measure[tw] OR measures[tw] OR findings[tw] OR result[tw] OR results[tw] OR test[tw] OR tests[tw])) OR generaliza*[tiab] OR generalisa*[tiab] OR concordance[tiab] OR (intraclass[tiab] AND correlation*[tiab]) OR discriminative[tiab] OR “known group”[tiab] OR “factor analysis”[tiab] OR “factor analyses”[tiab] OR “factor structure”[tiab] OR “factor structures”[tiab] OR dimension*[tiab] OR subscale*[tiab] OR (multitrait[tiab] AND scaling[tiab] AND (analysis[tiab] OR analyses[tiab])) OR “item discriminant”[tiab] OR “interscale correlation*”[tiab] OR error[tiab] OR errors[tiab] OR “individual variability”[tiab] OR “interval variability”[tiab] OR “rate variability”[tiab] OR (variability[tiab] AND (analysis[tiab] OR values[tiab])) OR (uncertainty[tiab] AND (measurement[tiab] OR measuring[tiab])) OR “standard error of measurement”[tiab] OR sensitiv*[tiab] OR responsive*[tiab] OR (limit[tiab] AND detection[tiab]) OR “minimal detectable concentration”[tiab] OR interpretab*[tiab] OR ((minimal[tiab] OR minimally[tiab] OR clinical[tiab] OR clinically[tiab]) AND (important[tiab] OR significant[tiab] OR detectable[tiab]) AND (change[tiab] OR difference[tiab])) OR (small*[tiab] AND (real[tiab] OR detectable[tiab]) AND (change[tiab] OR difference[tiab])) OR “meaningful change”[tiab] OR “ceiling effect”[tiab] OR “floor effect”[tiab] OR “Item response model”[tiab] OR IRT[tiab] OR Rasch[tiab] OR “Differential item functioning”[tiab] OR DIF[tiab] OR “computer adaptive testing”[tiab] OR “item bank”[tiab] OR “cross-cultural equivalence”[tiab])

NOT

("Address" [Publication Type] OR “biography”[Publication Type] OR “case reports”[Publication Type] OR “comment”[Publication Type] OR “directory”[Publication Type] OR “editorial”[Publication Type] OR “festschrift”[Publication Type] OR “interview”[Publication Type] OR "Lecture" [Publication Type] OR "Legal Case" [Publication Type] OR “legislation”[Publication Type] OR “letter”[Publication Type] OR “news”[Publication Type] OR “newspaper article”[Publication Type] OR “patient education handout”[Publication Type] OR "Popular Work" [Publication Type] OR "Congress" [Publication Type] OR “consensus development conference”[Publication Type] OR “consensus development conference, nih”[Publication Type] OR “practice guideline”[Publication Type]) NOT (“animals”[MeSH Terms] NOT “humans”[MeSH Terms]) NOT (("Child"[Mesh] OR "Adolescent"[Mesh] OR "Infant"[Mesh]) NOT "Adult"[Mesh]) NOT (bone sarcoma[ti] OR "Osteosarcoma"[Mesh])
